# A Web-Based Application for Complex Health Care Populations: User-Centered Design Approach

**DOI:** 10.2196/18587

**Published:** 2021-01-13

**Authors:** Francesca Ferrucci, Manuele Jorio, Stefano Marci, Antonia Bezenchek, Giulia Diella, Cinzia Nulli, Ferdinando Miranda, Guido Castelli-Gattinara

**Affiliations:** 1 Informapro Srl Rome Italy; 2 Department of Human Sciences, Communication and Tourism University of Tuscia Viterbo Italy; 3 EuResist Network European Economic Interest Grouping Rome Italy; 4 Unità Operativa Complessa Materno-Infantile - Azienda Sanitaria Locale Rieti, Consultorio Pediatrico Rieti Italy; 5 Academic Department of Pediatrics Division of Immune and Infectious Diseases - Istituto di Ricovero e Cura a Carattere Scientifico Ospedale Pediatrico Bambino Gesù Rome Italy

**Keywords:** patient, community participation, eHealth, patient-centered care, user-centered design, comorbidity

## Abstract

**Background:**

Although eHealth technology makes it possible to improve the management of complex health care systems and follow up on chronic patients, it is not without challenges, thus requiring the development of efficient programs and graphic user interface (GUI) features. Similar information technology tools are crucial, as health care populations are going to have to endure social distancing measures in the forthcoming months and years.

**Objective:**

This study aims to provide adequate and personalized support to complex health care populations by developing a specific web-based mobile app. The app is designed around the patient and adapted to specific groups, for example, people with complex or rare diseases, autism, or disabilities (especially among children) as well as Alzheimer or senile dementia. The app’s core features include the collection, labeling, analysis, and sorting of clinical data. Furthermore, it authorizes a network of people around the patient to securely access the data contained in his or her electronic health record.

**Methods:**

The application was designed according to the paradigms of patient-centered care and user-centered design (UCD). It considers the patient as the main empowered and motivating factor in the management of his or her well-being. Implementation was informed through a family needs and technology perception assessment. We used 3 interdisciplinary focus groups and 2 assessment surveys to study the contexts of app use, subpopulation management, and preferred functions. Finally, we developed an observational study involving 116 enrolled patients and 253 system users, followed by 2 feedback surveys to evaluate the performance and impact of the app.

**Results:**

In the validated general GUI, we developed 10 user profiles with different privacy settings. We tested 81 functions and studied a modular structure based on disease or medical area. This allowed us to identify replicable methods to be applied to module design. The observational study not only showed good family and community engagement but also revealed some limitations that need to be addressed. In total, 42 of 51 (82%) patients described themselves as *satisfied* or *very satisfied*. Health care providers reported facilitated communication with colleagues and the need to support data quality.

**Conclusions:**

The experimented solution addressed some of the health system challenges mentioned by the World Health Organization: usability appears to be significantly improved when the GUI is designed according to patients’ UCD mental models and when new media and medical literacy are promoted. This makes it possible to maximize the impact of eHealth products, thereby overcoming some crucial gaps reported in the literature. Two main features seemed to have potential benefit compared with other eHealth products: the modeling, within the app, of both the formal and informal health care support networks and the modular structure allowing for comorbidity management, both of which require further implementation.

## Introduction

### Background

The improvement in health services and the quality of health treatment and social care has led to a significant increase in survival (and quality of life) among adults and children with chronic complex diseases and high health care needs [1].

According to the World Health Organization (WHO), over a billion people have some form of disability, whereas 110 to 190 million adults have significant difficulty functioning. An estimated 39% of the Italian population is affected by some chronic disease, with increasing disability rates. Currently, more than 3 million people in Italy are disabled. These patients are characterized by multiple morbidities, requiring the use of a range of services and a technology-enhanced care model [1-4].

eHealth may help such patients manage multiple clinical encounters and large amounts of clinical information generated from various sources. Indeed, patients report a highly frequent use of information and communications technology (ICT) to search for health information, communicate with health care providers (HCPs), track medical information and medications, and assist in decision making regarding treatment [5]. Notably, patients attempt to use ICT tools for self-management, as they expect to benefit from eHealth and enhance control over their own disease [6].

Extant research suggests that eHealth tools supporting patient-HCP interaction, patient self-management, and HCP-HCP interactions (through electronic health record integration) are of great benefit to patients [7,8]. These benefits may increase further, as the COVID-19 crisis has triggered additional demand for remote care models and systems. Previous studies have pointed out a number of critical issues concerning complex health care populations, since these include different subpopulations that pose specific medical and organizational challenges for the design of public service provision. These issues include the accurate assessment of the levels of services and needs, implementation of services and resources tailored to specific needs, coordination and integration of family-centered care planning, promotion of health systems based on patient or family self-management, and the redefinition of models of multidisciplinary team care [5,9,10].

According to the 2012-2020 eHealth Action Plan, in 2011, the Italian Public Administration promoted a high-communication health care project and a citizen’s Electronic Health Dossier (*Fascicolo Sanitario Elettronico*) [8,11], but the project encountered difficulties in getting under way and proved difficult to implement. The few ongoing initiatives have not received positive feedback from users due to usability problems and the low digital literacy of both HCPs and families [12].

### Objectives

In this context, the ABILITA2 Project (Italian: *Sviluppo di un Applicativo per terminali moBILI dedicato a popolazioni ad alTA complessità Assistenziale*; English: *Development of a web-based Mobile Application for complex healthcare populations*) takes advantage of ICT and its eHealth applications, exploiting the patient-centered care approach. When addressing the abovementioned issues, it adapts the service to different subpopulations, providing models that can be replicated in the future [13].

To meet the requirement of interdisciplinarity, the ABILITA2 consortium includes a partnership between ICT companies (Informapro Srl, Logica Informatica Srl, and Mediamed Interactive Srl) and medical and research centers (*Ospedale Pediatrico Bambino Gesù* - Rome and *Consultorio Pediatrico* ASL Rieti) as well as patient associations related to the medical areas of Alzheimer disease, autism, artificial nutrition, and rare pediatric diseases.

The project’s general objective was to provide adequate and personalized support to complex health care populations by developing a specific web-based app, *Abilita*, designed around the patient and customizable for specific groups, notably people with complex or rare diseases (eg, genetic syndromes, patients requiring parenteral nutrition), autism or disabilities (especially among children), and Alzheimer or senile dementia. The core features of the app allow for the collection, labeling, analysis, and sorting of clinical data. Furthermore, it authorizes a network of people around the patient to securely access the data contained in his or her electronic health record.

The study’s specific objectives are as follows:

- Assess levels of service and patient needs, testing assessment procedures and tools, especially for pediatric and older adult groups who are less considered in the eHealth market.
- Promote patient self-management and co-responsibility as the basis for a suitable and user-friendly web application. The emphasis is on patient empowerment (understanding of his or her role, acquisition of sufficient knowledge to be able to engage with HCPs, patient skills, and the availability of a facilitating environment [14,15]).
- Enhance and innovate the coordination between professionals and caregivers, specifically exploring the potential of a collaborative network operating on the patient’s behalf, which is built by the patient based on his or her individual needs and institutional contacts.
- Make the most of a *proximity support network*, which includes informal relationships with relatives, friends, and key figures in the territory, which is a crucial health care management factor [16,17].
- Encourage families or communities to play an active role and, at the same time, ensure quality of data, care, and assistance by using GUI modeling of proper actions per profile according to the level of skill and motivation.
- Assess the app’s performance and impact.

## Methods

### Assessment and Design Process

The project adopted a user-centered design (UCD) approach in graphic user interfaces (GUIs) and considered users’ point of view and needs as central. The difference from other methods is that UCD meets the needs and desires of users rather than forcing them to change their behavior to meet the product settings [18]. Since the designers considered the user to be the patient (or parent/caregiver), an interdisciplinary analysis was needed to assess needs and then model actions, logic paths, questions, and answers within the interface. To do so, clinical and medical competence needs to be flanked by skills in computer sciences and database management, communication or new media sciences, psychology, and sociology [13]. The study used a number of focus groups based on a general inductive approach. The results of these focus groups were then further investigated through anonymous questionnaires [19]. The focus groups met monthly with 90- to 120-min sessions to analyze the different issues raised by the study.

Focus group A assessed patients’ needs and scenarios of use. It included patients (n=4), health care workers (n=2), psychologists (n=1), researchers in communication sciences (n=1), and software developers (n=1). All participants were part of the project network and discussed the experience of patients and caregivers with ICT products and possible scenarios using the Abilita app. Finally, a web-based questionnaire (Q1) was developed for the purpose of studying the main features, habits, needs, and digital and medical literacy of patients and families. Q1 was sent to a selected sample of patient associations (presidents and expert members in steering groups): *Alzheimer Uniti Roma ONLUS, Associazione Nazionale Genitori Soggetti Autistici (ANGSA) Lazio Onlus, Associazione italiana sulla nutrizione Artificiale Domiciliare “Un filo per la Vita,” Associazione Prader Willi Lazio, Associazione Italiana delezione cromosoma 22 Onlus*. The 20 anonymous responses were collected in June 2018; and the statistics of multiple-choice items and summaries of open-answer items were contained in a project report in September 2018 [20,21].

Focus group B, consisting of HCPs (n=4), psychologists (n=1), privacy officers (n=1), and software developers (n=2), was devoted to the general GUI design. The outcomes of the assessment of patient needs were translated into design challenges. The discussion raised a number of research questions, including the problem of low HCP motivation or time and the need to consider the patient as the main subject motivated to use the app. It is also necessary to task the patient or caregiver with data entry and updating health records and adding user profiles to the app (to model both institutional and informal patient support networks). Additional issues concerned the powers of individual user profiles (reading or writing of sections of the data set), the need to ensure health data quality, even when not directly entered by HCPs, and to predict real-world data entered by the patient and his or her proximity network. We used paper prototyping throughout the process that led to the user requirements document delivered in November 2018 for all identified user profiles (patient, parent or tutor, caregiver, family member, doctor, nurse, structure manager, social operator, temporary, and emergency).

In designing the health record, we tried to identify possible user behaviors, which led to additional questions: what does a particular population require and how can the interface structure be customized for specific pathologies to meet patient needs and coordination requirements? Data and pages are not equally relevant for all subpopulations, and preferred content, information, and functionalities differ across groups. In this respect, the general GUI of *Abilita* could be made more powerful by customizing content and database structure, with a view to create GUIs for more specific medical areas (the Abilita *modules*).

Focus group C was set up to assess this potential. It included presidents and steering group members from patient associations (n=4), psychologists (n=1), communication sciences researchers (n=1), and software developers (n=1). The discussion addressed the specific needs of the subpopulations involved in the study, after which we administered a mandatory questionnaire (Q2) to test the usefulness and effectiveness of feasible implementations. Q2 was sent out through email to a selected sample of national and regional patient associations; the 15 anonymous responses were then collected into a database highlighting the main aspects or attention points for GUI customization and the preferred functions that could be identified.

### Observational Study, Feedback, and Validation

After the development of the prototype, we performed an observational study to evaluate its application in terms of its functionality, versatility, responsiveness to patients or families’ needs, user-friendliness, and rate of acceptance. We designed the study in line with international Good Clinical Practice criteria and obtained approval from the ethics committees of the medical centers involved (document protocols 1589_OPBG_2018 and 2474/CE Lazio1).

A total of 116 of the 130 (89.2%) patients invited to participate in the study were included, as they (or their families) possessed the required computer skills. They were recruited in the Rome area and in the Province of Rieti, a setting marked by a variety of health needs and increased geographic isolation due to the 2016 earthquake. During the 6-month study period (January-June 2019), the patients authorized additional user profiles to access their data, namely 32 HCPs, 97 parents, 5 family members, and 3 caregivers, for a total of 253 app users.

We then analyzed individual user accesses to explore the actual use of the app. Frontal, telephone, and web-based tutoring sessions helped the patient participants (or their parents if the patient was aged under 16 years) to complete the registration and browse the app upon uploading their personal data. In June 2019, we developed a voluntary web application feedback questionnaire for patients (Q3) with indicators for evaluating usefulness or satisfaction, privacy, and security impact. We identified usability and effectiveness, while task managers tested the app’s compliance with general recommendations and technical functionality. A link to the questionnaire was sent by email (we avoided multiple responses by limiting survey access to a single instance), and we received 51 anonymous responses in July 2019; the statistics on multiple-choice items and summaries of open-answer items were reported in a project report in September 2019.

In July 2019, we conducted 23 semistructured individual interviews with 10 doctors and 13 nurses to explore the app’s usefulness in the follow-up of chronic patients, its usability, and other features of the HCP interface (questionnaire Q4).

Table 1 summarizes the different data collection stages of the research.

**Table 1:** Data collection processes.

Data collection process	Description	Access and recruitment criteria	Collected data and period	Output
Focus group A	8 participants (4 members of the patients’ associations or caregivers, 2 HCPs^a^, 1 software programmer, and 1 psychologist); 1 facilitator (researcher in communication sciences)	Members of the project network, experienced in the management of 5 medical areas (autism spectrum disorders, 22q11.2 deletion syndrome, Alzheimer disease, Prader-Willi syndrome, chronic intestinal failure)	Eight 2-hour meetings in the period, April-May 2018	Definition of main aspects and attention points to be tested on a larger sample of respondents through the questionnaire Q1; definition of scenarios of use
Questionnaire Q1	62 items mostly in a multiple-choice format and with partial adaptative questioning	A web questionnaire mandatory for a restricted sample of national and regional patient association members (closed mandatory survey [21])	20 anonymous responses collected in May 2018	Project report
Focus group B	8 participants (2 software programmers, 2 doctors, 2 nurses, 1 psychologist, and 1 privacy officer); 1 facilitator (researcher in communication sciences)	Members of the project network, experienced in eHealth and GUI^b^ design processes	Fifteen 2-hour meetings in the period, June-November 2018	User requirement document for all the identified user profiles
Focus group C	6 participants (4 members of the patients’ associations, 1 software programmer, and 1 psychologist); 1 facilitator (researcher in communication sciences)	Members of the project network, experienced in the management of 5 medical areas (autism spectrum disorders, 22q11.2 deletion syndrome, Alzheimer disease, Prader-Willi syndrome, chronic intestinal failure)	Five 2-hour meetings in the period, December 2018-January 2019	Definition of main aspects and attention points to be tested on a larger sample of respondents through the questionnaire Q2
Questionnaire Q2	7 items mostly in an open-answer format	Text file sent by email to a selected sample of national and regional patient association members (closed mandatory survey [21])	15 anonymous responses collected in January 2019	Database with main aspects and attention points for customization of the GUI
Observational study	Use of the *Abilita* app in real-world settings by patients, families, HCPs, and communities	We invited 130 patients of the project medical centers to participate (Provinces of Rome and Rieti); 116 accepted the invitation and were recruited	253 system users in the period January-June 2019 (116 patients, 32 HCPs, 97 parents, 5 other family members, and 3 caregivers)	Report on statistics of use in real-world settings exported by the system administrators
Questionnaire Q3	36 items mostly in a multiple-choice format (16 defined by a Likert scale score) and with partial adaptative questioning	A web questionnaire; we invited the 116 patients involved in the observational study and obtained 51 responses (closed voluntary survey [21])	51 responses collected in July 2019	Project report
Questionnaire Q4	17 items (16 defined by a Likert scale score and 1 open-answer item)	Face-to-face interviews; we invited the 32 HCPs involved in the observational study; 23 accepted	23 responses collected in July 2019	Project report

^a^HCP: health care provider.

^b^GUI: graphic user interface.

## Results

### Assessment and Design Process

Q1 clarified the overall context of the study. The age at first diagnosis for complex health care diseases ranged from 0 to 5 years for the majority of cases and from 65 to 80 years in the remaining cases. All patients were not autonomous and had at least one caregiver. Their digital skills were at a basic or medium level, with limited experience with the use of IT tools to communicate with social and (private or public) health care services. Patients or caregivers displayed significant awareness of their medical areas. They were able to name the diagnosis in technical terms, describe the main elements of the disorder or disease (causes, severity, symptomatology, correlations with other disorders, and risk factors), mention the pharmacological therapies with precision, describe recommended daily treatments and activities (diets, sport), and recognize changes in symptoms (especially aspects to be monitored and reported to health care personnel). The most frequently used documents were treatment plans, reports of visits or exams, and prescriptions. Most patients reported to a health care unit devoted to their specific disorder or disease and scheduled follow-up visits every 6 months on average. In this context, potential clients believed that *Abilita* could successfully respond to the following requirements:

- Provision of tools and resources to manage emergency situations (average score of 8.2 on a 0-10 scale, SD 1.6).
- Collection and storage of health care documents and digital contents (average score of 7.7 on a 0-10 scale, SD 3.0).
- Remote communication with authorized health care personnel (average score of 7.6 on a 0-10 scale, SD 2.1).
- Support with monitoring activities (reminders of exams, visits, self-measurements, etc; average score of 6.7 on a 0-10 scale, SD 2.8).
- Targeted information on recreational, informative, or social activities (average score of 6.1 on a 0-10 scale, SD 2.3).

Focus group A identified the Online Help function as a central tool for the app, as it served multiple goals: it accompanies the user in browsing the sections even when he or she has low digital or medical literacy, and it acts as an intermediary between the different users operating within a patient’s personal folder.

**Figure 1:**
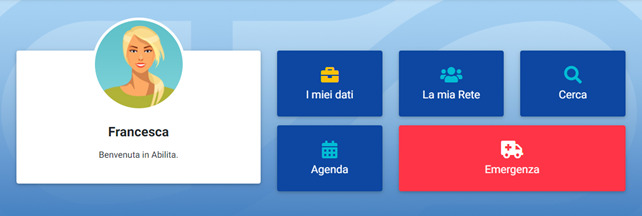
Home page–shortcuts to the main areas and welcome or follow-up message from Lisa.

Focus group B confirmed the main areas of the GUI (menu items) as follows: *Home page; Help; My data; My network; Search; My story; Organizer; Notifications; Personal profile; Info room; Emergency card*. The Online Help, personalized as a female avatar named *Lisa*, interacts with the user by written and/or audiovisual messages. The app also features a medical glossary explaining technical terms and jargon. When users first access the app, *Lisa* provides advice and recommendations on how to start, suggests the sections to be prioritized, and offers easily accessible demos of app functions. In subsequent usage, *Lisa* highlights unread notifications, scheduled appointments, and missing information in the Emergency card when relevant (Figure 1).

**Figure 2:**
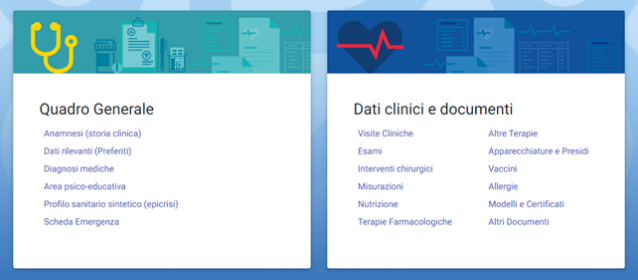
Area “My data.”.

The *my data* area is the medical and administrative record and comprises 2 sections: *general outline* and *clinical data and documents* (Figure 2). The sections include *importance or severity* labels that ensure the record’s organization and facilitate access to the most relevant data. Key information on the type of disease, therapy, particular care needs, and specific conditions is easily available. Thanks to the *validation* function, HCPs can validate data entered by patients or caregivers.

**Figure 3:**
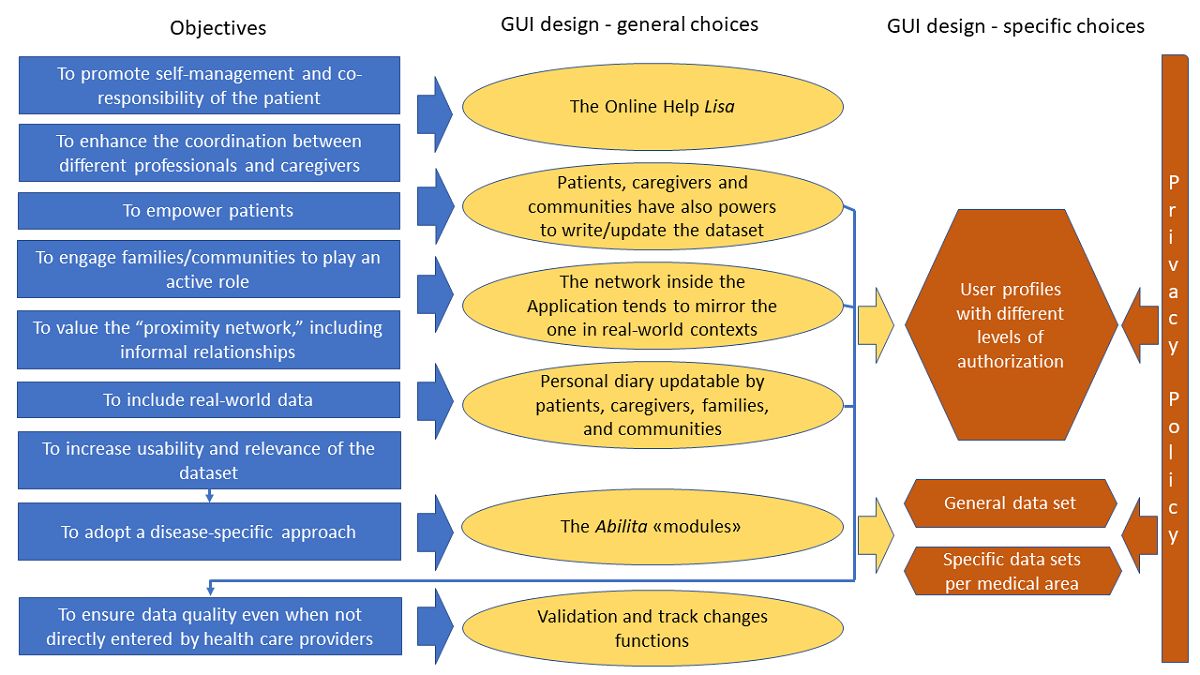
The design process.

In the area *my network*, the patient or the parent or legal tutor can create a personalized collaborative network of care support (eg, doctors, nurses, parents, friends, neighbors, domestic helpers, babysitters and tutors, teachers, etc). Each member of the network is assigned a separate profile with authorization to access some or all of the personal data. Furthermore, the patient may authorize all health care facilities, thereby enabling all HCP personnel to read and update their medical records. The app also makes available temporary or emergency authorization facilities as well as the blanket withdrawal of all permissions. In the *search* area, it is possible to carry out simple or advanced database searches sorted by data subject or by authorized person (highly recommended by HCPs to facilitate access to relevant information). *My story* hosts a personal diary where users can note clinical data as well as daily experiences, relevant episodes or therapeutic adherence (Multimedia Appendix 1). Actions in the app are always traceable, which allow reconstruction of the author and the date of changes and data validation. Figure 3 summarizes the results of the design process, the relationship between the design and objectives of the research (as discussed in the focus groups) and privacy policy.

In keeping with the privacy policy, the patient is the sole owner and controller of his or her data and the only person able to decide who may treat them and under what conditions, which meets both General Data Protection Regulation requirements and recommendations concerning patient empowerment [22,23]. All sensitive data and interactions between the client (web-based application or emergency mobile app) and the server are encrypted.

The results of focus group C confirm that the GUI’s disease specificity crucially improves app usability and patient engagement. The relevance of the data set and the perception of utility by families and communities increases when the app is customized based on the specific needs of a subpopulation. In particular, we studied subpopulation management for the following medical areas: autism spectrum disorders, 22q11.2 deletion syndrome, Alzheimer disease, Prader-Willi syndrome, and chronic intestinal failure. The main gaps were centered around the coordination of social and health care services (mostly during follow-up) as well as family support. As a result, the design of the *Abilita* *modules* for each medical area includes specific GUI features: personalization of the content and structure of the medical data set, contents of the *info room* (information about the disease), and functions of the *organizer* and *notifications* as well as recommendations and priority highlights from Lisa. More specifically, the study foregrounded the following elements:

- Each subpopulation would like to have a personalized page in the *clinical data* subsection.
- Different diseases and ages need differentiated administrative forms.
- The agenda and remind functions could be implemented for specific situations and connected with local networks.
- Users consider it important that data for clinical research at different levels be available.
- Users consider the latest disease-specific documents and recommendations important, such as the Integrated Care Pathway or best clinical practices.

### Observational Study, Feedback, and Validation

Table 2 shows the characteristics of enrolled patients and families as well as their average use of the *Abilita* app over the last 4 to 6 months of study. These data were automatically exported by the system administrators and reflect the actions performed by users within the app, including demographic data entered at registration.

Owing to the characteristics of the investigators (pediatricians), most of the enrolled subjects were children or adolescents, in which case the users of the app were mainly parents or family members. HCPs authorized by patients or parents primarily uploaded clinical data and documents. Patients performed operations such as consultation with clinical data, loading of missing clinical investigations, and writing of individual day-to-day experiences. Each patient authorized an average of approximately 2 persons to access their data, who were usually parents and family members, doctors, nurses, and psychologists. By contrast, caregivers and school operators were considerably less involved. The 868 documents that were uploaded included 18 different subtypes, mainly reports of examinations and clinical investigations. Approximately 35% of the data entries were performed by the patients or their parents from the beginning.

We tested 81 *Abilita* functions, which users could access with different levels of authorization (Multimedia Appendix 2). Q3 involved 51 respondents. Table 3 shows the results of the answers to questions 1 to 16, with average positive scores of 78% (4 or 5).

**Table 2:** Statistics of use of the study population (N=116).

Parameters		Participants
Males, n (%)		67 (57.8)
**Age (years), n (%)**		
	0-10	67 (57.8)
	10-20	28 (24.1)
	>20	21 (18.1)
Accesses by patients (n=623), mean (SD)		5.4 (2.3)
Authorizations by patients, n		207
Entered documents, n		868
Entered clinic visits, n		307
Entered exams, n		271
Entered diagnoses, n		155
Entered vaccines, n		348
Entered inputs on importance or severity, n		1040
Authorized parents, n		97
Other authorized family members, n		5
Authorized caregivers, n		3
Authorized HCPs^a^, n		32

^a^HCP: health care provider.

**Table 3:** Answers to questions 1-16, expressed in percentage of Likert scale scores.

Question No.	Question	Scores, n (%)		
		1 or 2	3	4 or 5
1.	Is *Abilita* useful for the orderly archiving of medical documents?	1 (2)	6 (12)	44 (86)
2.	Is *Abilita* useful for the orderly archiving of documents concerning care and assistance?	2 (4)	6 (12)	43 (84)
3.	Is *Abilita* useful for remembering the renewal of some clinical evaluations?	0 (0)	10 (20)	41 (80)
4.	Is *Abilita* useful to having your medical history under control everywhere?	1 (2)	2 (4)	48 (94)
5.	Does *Abilita* allow you to monitor some medical parameters when recommended by the HCPs^a^?	2 (4)	7 (14)	42 (82)
6.	Is *Abilita* useful for recording daily self-measurements (eg, blood pressure)?	10 (20)	9 (18)	32 (62)
7.	Does *Abilita* allow you to share information on healthcare or psycho-educational assistance with various professionals?	3 (6)	8 (16)	40 (78)
8.	Does *Abilita* allow you to receive relevant information in a health emergency away from home?	0 (0)	6 (12)	45 (88)
9.	Does *Abilita* allow you to share health information with HCPs without bringing your complete medical chart with you?	0 (0)	3 (6)	48 (94)
10.	Does *Abilita* help you adhere to drug therapy regimens (with reminders) and track what has actually been taken?	6 (12)	10 (20)	35 (68)
11.	Does *Abilita* help you remember which medical devices to buy or order?	6 (12)	14 (27)	31 (61)
12.	Does *Abilita* help you remember administrative deadlines for requesting disability status or for other socio-healthcare procedures?	5 (10)	13 (25)	33 (65)
13.	Does *Abilita* help you to find a document in your archive quickly using advanced search functions?	3 (6)	8 (16)	40 (68)
14.	Does *Abilita* provide useful information about bureaucratic aspects, scientific research or treatments?	2 (4)	14 (27)	35 (69)
15.	Can *Abilita* support HCPs in drawing up a treatment plan and help you follow it?	2 (4)	10 (20)	39 (76)
16.	Overall were you satisfied with the trial run of *Abilita*?	1 (2)	8 (16)	42 (82)

^a^HCP: health care provider.

Questions 17 and 18 asked users about the areas they would like to see enhanced: the answers covered all the areas suggested, with no specific option prevailing significantly, and the same applies to what functions should be integrated (question 18). Interestingly, the option *ability to set preferred tabs or activities to create shortcuts for most used functions* obtained 37% (19/51) of the responses, suggesting that customization is the best strategy. No relevant issues arose regarding privacy and security (questions 19-20): 57% (29/51) of users had no general problems, 65% (33/51) had no problems entering and classifying data, only 23% (12/51) had problems but overcame them with the Lisa online help or with practice (questions 21-30).

Other open and unstructured optional questions (31-36) yielded good feedback concerning the Lisa web-based help, with 47% (24/51) suggesting further implementation of this tool. Patients and caregivers urged informing family doctors and pediatricians about the app to maximize dissemination. The answers on scientific research and on PDTAs (diagnostic-therapeutic assistance pathways) highlight *Abilita*’s potential for data collection subject to privacy consent, for reconstructing analogies in groups of patients affected by the same disease or disorder, and for patient associations to pursue their institutional goals. In addition, *Abilita*’s effectiveness in facilitating relationships or communication with HCPs and local facilities was positively evaluated, preferably with the support of the region. Furthermore, participants considered that the main strengths of the project were *knowledge of one's own medical history with a click* and the overall philosophy behind the app (Multimedia Appendix 3).

Q4, which included 17 predefined questions and addressed 23 HCPs, produced average positive scores of 72% (4 or 5) in the first 16 items defined by a Likert scale score (Multimedia Appendix 4). In the last open-answer item, asking strengths or weaknesses of the project, the following aspects were highlighted:

- The availability of reports and alerts facilitated communication among HCPs and accelerated diagnostic and care paths.
- Users appreciated the involvement of patients or parents in the data entry of documents, lab results, and parameters, although 6 respondents raised concern about quality.
- Overall, 39% (9/23) of respondents encountered general problems in using *Abilita*, especially in the first weeks, and asked that Online Help tools be implemented.
- Users appreciated the importance or severity labels.

## Discussion

### Principal Findings

The project used needs assessment to establish the contexts to interface with, showing a prevalence of non–self-sufficient patients—typically infants and older adults—diagnosed at an average age of 0 to 5 or 65 to 80 years and mainly supported by health care units specifically devoted to the disorder or disease, for whom follow-up visits are scheduled on average every 6 months. Basic digital skills and good levels of medical literacy of families were identified as starting points of the design.

A sample of 116 patients participated in the observational study. Each patient authorized an average of 1.8 persons to access his or her data, typically parents and family members, doctors, nurses, and psychologists, with the additional involvement of the communities of other institutions and informal environments, for a total of 253 system users. In approximately 35% of cases, data entry was performed by the patients or their parents from the beginning.

Questionnaire Q3 yielded positive patient feedback on the utility of the app to address some health system challenges mentioned as relevant by WHO [24] and on themes such as delayed reporting of events (WHO challenge 1.2), communication roadblocks, lack of access to information or data, insufficient utilization of data and information (WHO challenges 1.4-1.6), insufficient continuity of care, inadequate supportive supervision (WHO challenges 3.5-3.6), low adherence to treatments, and loss of follow-up (WHO challenges 5.2-5.4).

We received no direct evidence on other challenges mentioned by WHO, such as low health worker motivation (3.4), geographic inaccessibility (5.2), insufficient patient engagement (8.1), or absence of community feedback mechanisms (8.3). Some useful indications do emerge in the interpretation of the answers to the same questionnaire Q3. The app promoted communication and team management among HCPs, health care bodies, and families (question 34) and, in addition, increased end user confidence in their own capacity to provide up-to-date, readily searchable, and clear medical information (question 36). According to answers to questions 33 and 35, *Abilita* can contribute to scientific research and PDTA definition (diagnostic-therapeutic assistance pathways), thereby addressing the lack of population denominator (challenge 1.1) —that is, once used by a larger sample of patients in the same medical area, it can become a tool for further assessment of subpopulation management.

The general choices of the GUI design revealed some advantages:

- The GUI is designed around the patient, who is modeled as the main empowered and motivating actor of the actions necessary to maintain and update the medical record.
- Users are constantly supported by the Online Help (avatar *Lisa*), thus addressing medical and digital literacy issues and patient’s commitment in terms of his or her specific role, the main problems that arise while using many ICT products.
- Coordination and management needs can be modeled as pathways and actions recommended by Lisa within the app; they are also addressed by targeted functions (search, calendar, and notification areas).
- Real-world data can be traced and collected to then be reused to advance research on the management of complex chronic conditions.

The issue of data quality, indeed highlighted by 6 of the respondents to the HCP survey, was addressed in the project through the track changes and validation functions. It is worth noting that patients and families are increasingly being required to participate in health monitoring, through daily self-measurement and recording of symptoms or in questionnaires, for diseases such as diabetes, and most recently in the COVID-19 pandemic [25,26]. eHealth market engagement strategies—especially in light of the new patient co-responsibility paradigm—are based on flexibility and customization, with a user-friendly design that makes it possible to communicate with or forward information or data to HCPs [27]. In its adoption of these strategies, *Abilita* is in line with a reframed relationship between active citizens and professionals and is intended as a social innovator in the development of a smart community model with the involvement of the proximity network–the app’s core feature.

Although informal or territorial networks were not fully exploited by the users during the observational study, as suggested by the number of authorized user profiles (Table 2), we can hypothesize that this was influenced by the study’s short duration and the characteristics of the patients involved, mainly children and teenagers. The lockdown period in Italy and Europe revealed the need to innovate public health systems precisely in this direction, linking them to local support networks (through new professional figures such as community nurses) and moving toward an integrated vision of health care. The role of volunteering and associations in providing support to self-isolated and vulnerable persons has also been highlighted [28,29]. In this context, specific design choices may require further refinement, considering, for example, the addition of other user profiles such as territory medicine physician or volunteer.

The modular structure of *Abilita* allows for the personalization of data sets and functions. It also facilitates far-sighted and sustainable investments owing to the partnership’s commercial initiatives, which are aimed at developing new modules (optimal feedback has already been received from relevant stakeholders) and intercepting specific target audiences interested in them. Most importantly, this structure allows the patient to choose one or more application *modules* in the case of different pathologies. In this way, *Abilita* has the added value of comorbidity management that is crucial to complex health care populations.

Usability appears to be significantly improved when the GUI is designed according to patients’ mental models and when new media and medical literacy are promoted. Following this principle, the assessment of specific subpopulation needs and the development of personalized GUIs for specific medical areas appears important. Procedures to assess patients’ needs were successfully experimented and a replicable methodology was defined.

### Limitations

This analysis was limited by the low number of enrolled subjects and its short duration. Data collected during the study period and answers to questionnaire Q3 refer mainly to pediatric populations; more evidence is needed about older adult patients’ feedback. In fact, only one quarter of them were adults or seniors, but the app was designed and particularly valid for non–self-sufficient subjects, both children and older adults.

The strategy of modular implementation appears to be the best one, but no module has yet been developed and tested. A complete comparison with other available apps, mainly focused on a single disease, will be relevant once the corresponding modules are developed. Specific GUI design choices need to be refined. Nevertheless, the study shows the versatility of this approach for complex health care populations.

### Conclusions

eHealth technology allows better management of complex health care aspects in the follow-up of chronic complex disease patients, but translating the UCD into GUI features of an eHealth app is a difficult task. The decision to use patient self-management and co-responsibility as the basis for an eHealth information system seems to have been successful in enhancing the probability of matching the needs of the target population. Moreover, usability appears to be significantly improved when the GUI is designed according to patients’ UCD mental models and when new media and medical literacy are promoted. Its potential applications in an era of greater sociosanitary distancing are certainly of particular interest.

Possible lines of exploitation are as follows:

- Design and develop new *Abilita* *modules* dedicated to specific clinical areas with particular care needs (not least with automatic data download and information managed by the patient’s clinical facility of reference).
- Make *Abilita* an integral part of the automatic distribution of data and dissemination of procedures in the public sector (The Italian National Health Care system is structured by regional area, with disease-specific health care facilities that may be very distant from users).
- Strengthen and expand *Abilita* and the patient association network to share information and solutions to the various problems faced by caregivers on a daily basis.
- Simplify usability as much as possible with the possible introduction of voice command shortcuts.

## Appendix

Multimedia Appendix 1 Screenshots and description of the Italian graphic user interface.

Multimedia Appendix 2 Tested Abilita functions.

Multimedia Appendix 3 Feedback questionnaire—patients.

Multimedia Appendix 4 Feedback questionnaire—health care providers.

## Abbreviations

GUI: graphic user interface
HCP: health care provider
ICT: information and communications technology
UCD: user-centered design
WHO: World Health Organization
