# Supplementary material for: A Web-Based Application for Complex Health Care Populations: User-Centered Design Approach
Source: JMIR Hum Factors. 2021 Jan 13;8(1):e18587. doi: 10.2196/18587 (PMC7840279; doi:10.2196/18587)

ABILITA2 PROJECT

Screenshots and description of the Italian GUI

Home page – shotcuts to the main areas and welcome/follow-up message by LISA

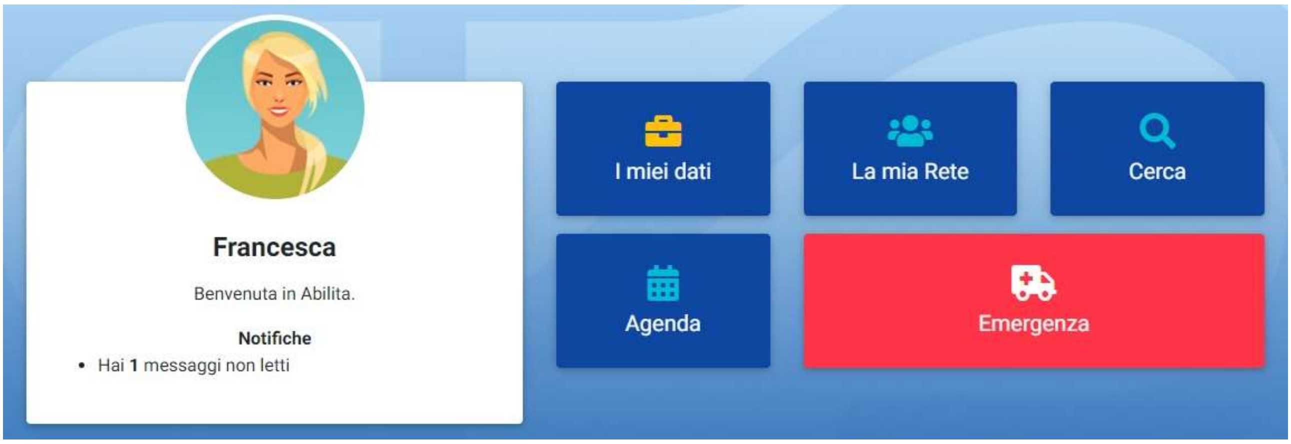

Area “Help” – Demo by LISA

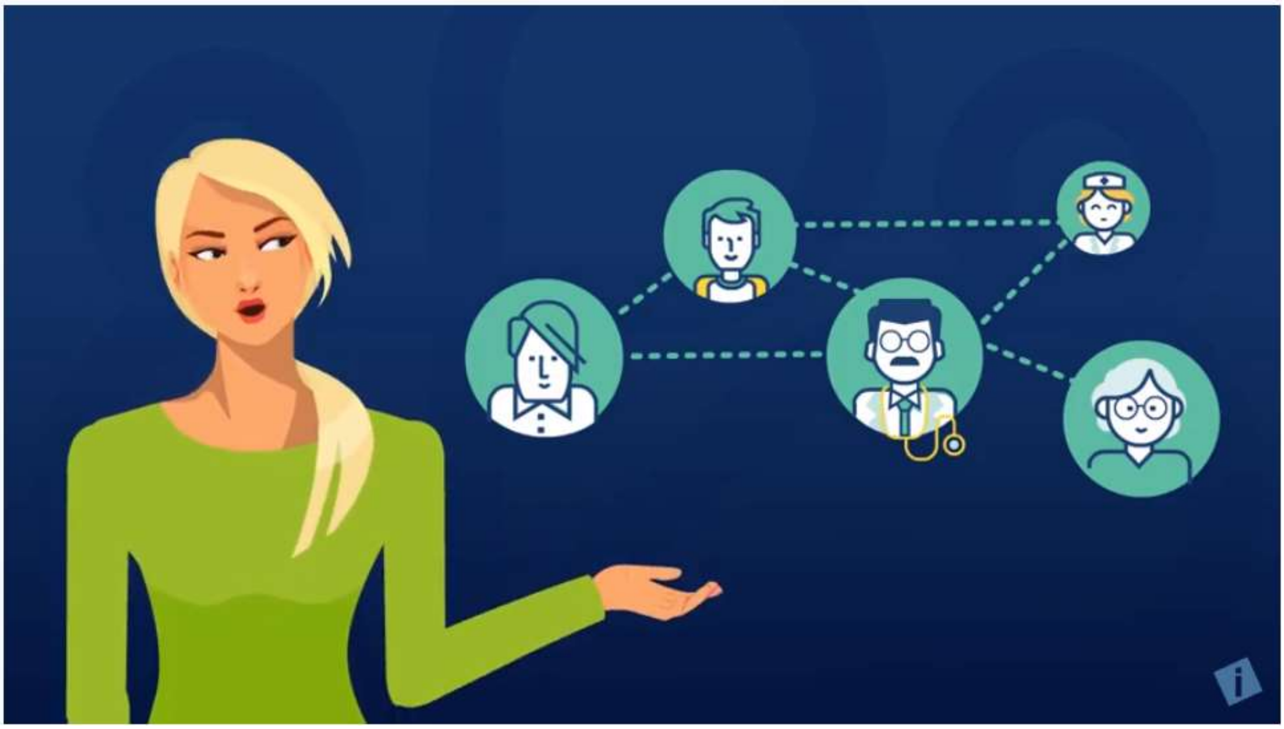

For all LISA communications, either in demos or written messages, readability tests have been performed, targeting a maximum of “medium” difficulty.

Area “My data” - overview

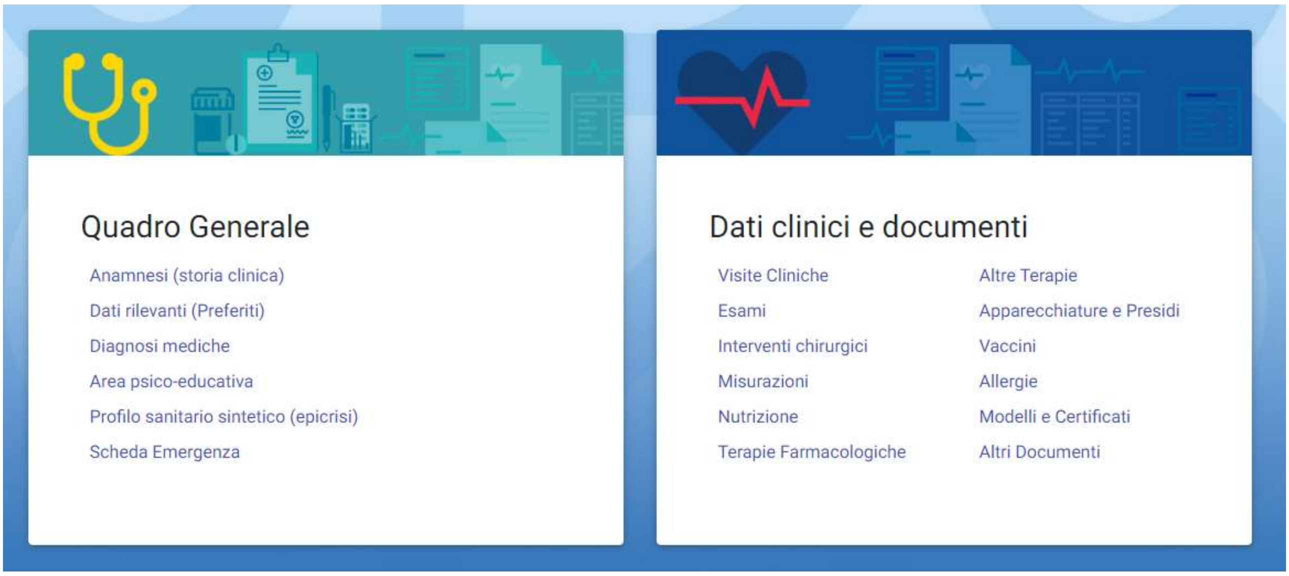

The area “My data” is the medical and administrative record, consisting of 2 sections, “General outline” and “Clinical data and documents”. The first section is thought of as the start of the patient and doctor involvement, where personal traits and relevant medical data are easy to display in “one click”. The family doctor or the case manager should be involved in the completion of this section. The second section should contain in-depth clinical information, following medical examinations or self-evaluation and measurements. Many sub-sections are also included to assist management of medical administrative documents and medical devices (for example, a reminder to order a special device and the related code).

Area “My network” – authorizing a healthcare structure to access my data

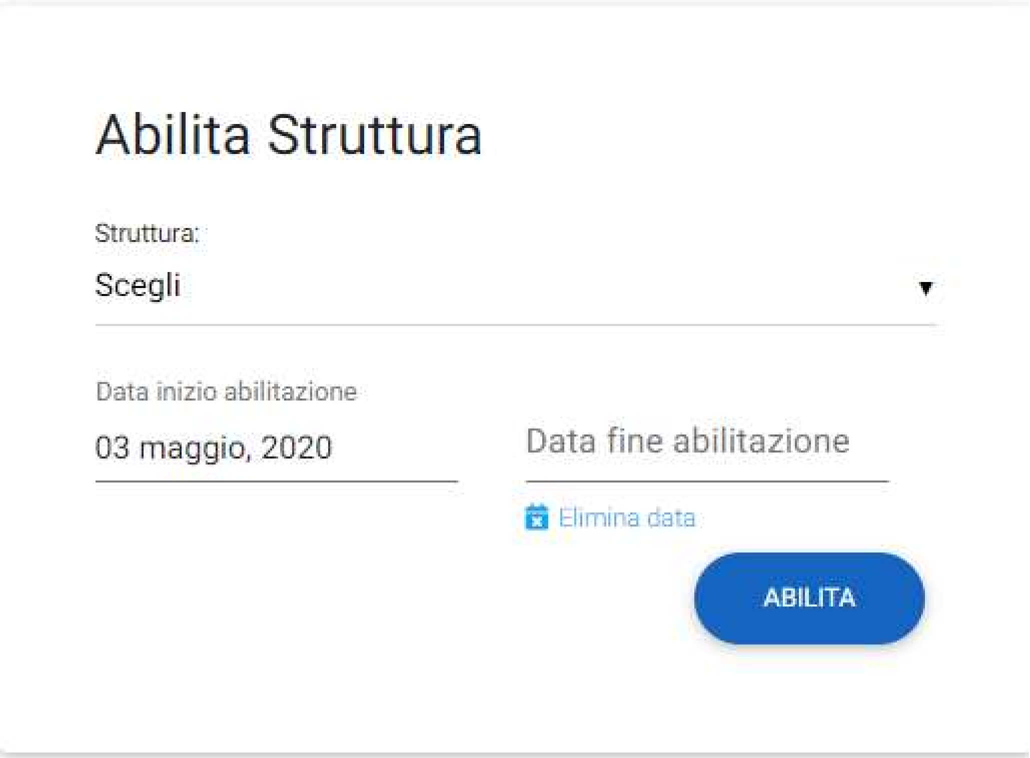

Area “My Network” – on demand written help message by LISA

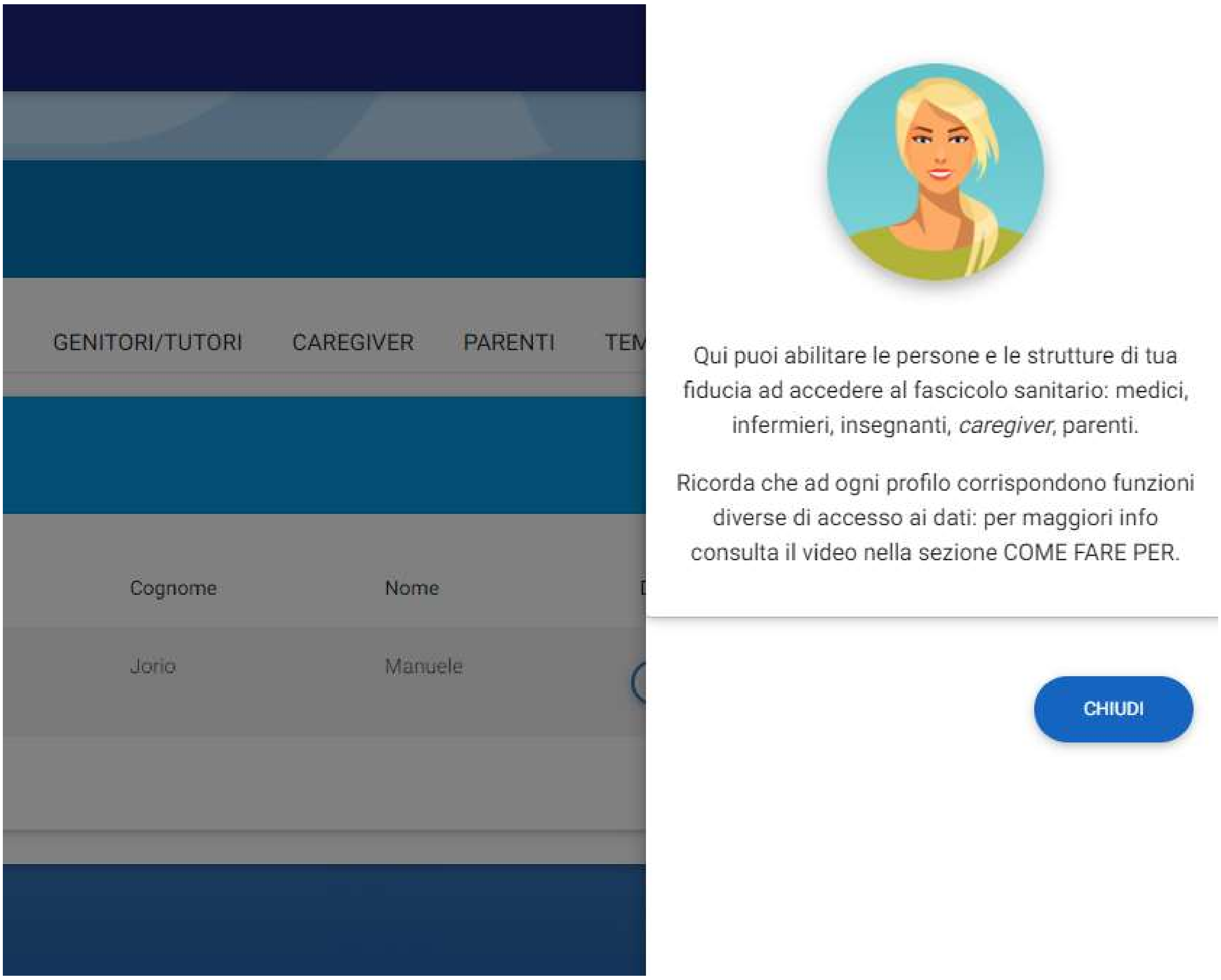

Area “Search”

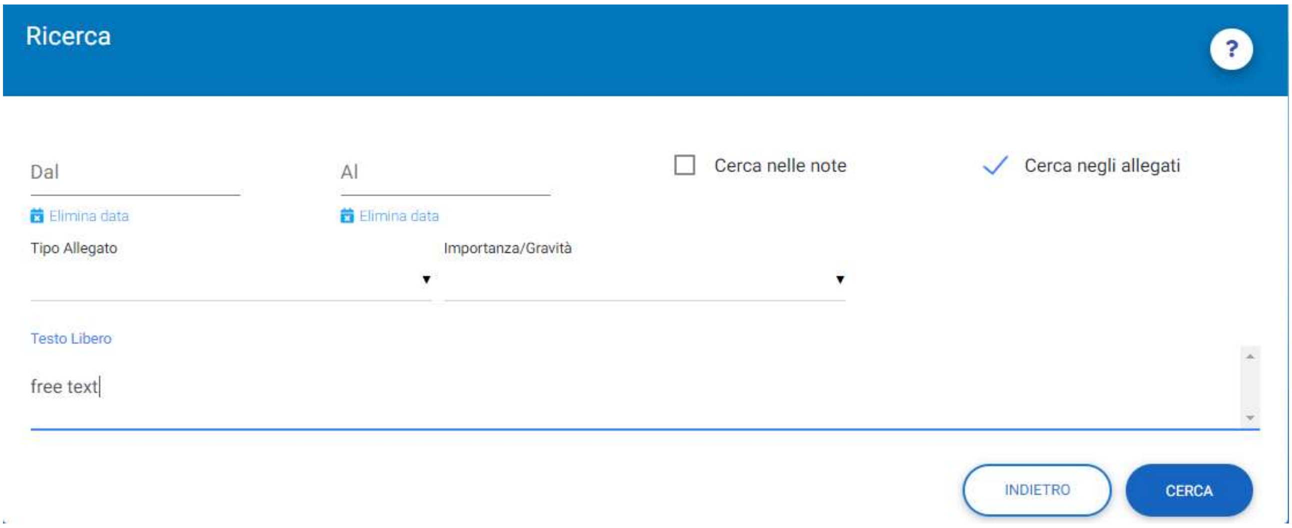

Area “My story” – inserting a new post of my diary

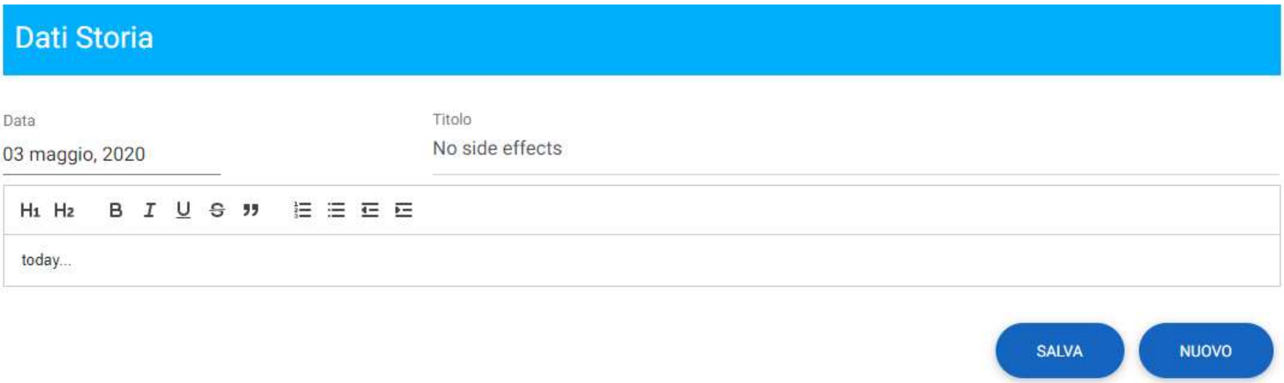

The “organizer” and “notifications” sections provide management services and help deal with appointments, duties and deadlines.

Organizer – setting the reminder for my visit

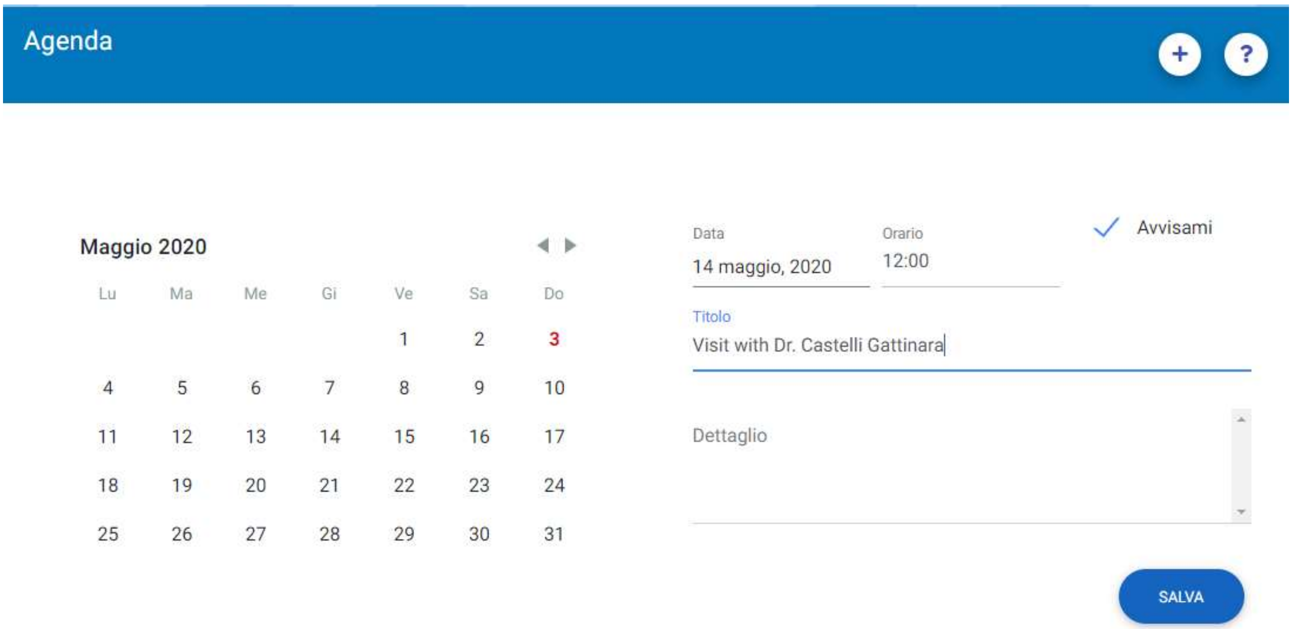

Notifications – reading my notifications

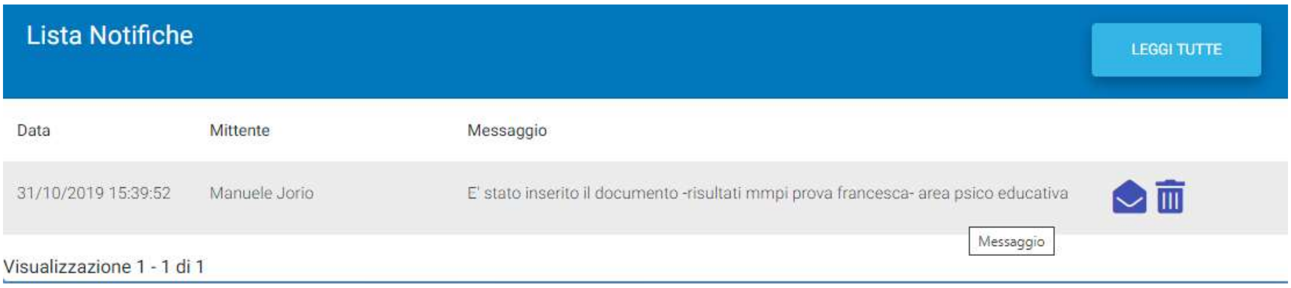

The “personal profile” summarizes the main inserted personal data, while “info room” gives practical information or guidelines for disabled people, complex healthcare populations and their families.

Inforoom – overview

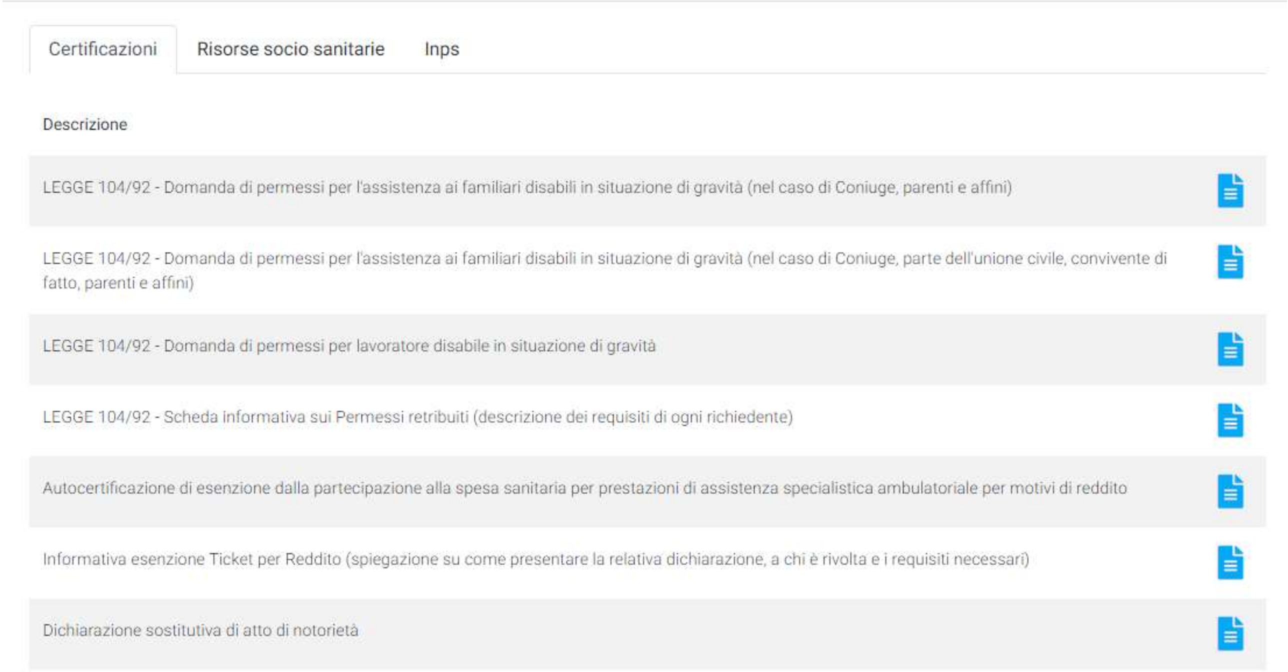

The Emergency card summarizes the relevant information for emergency situations.

Emergency card – detail of 4 sections

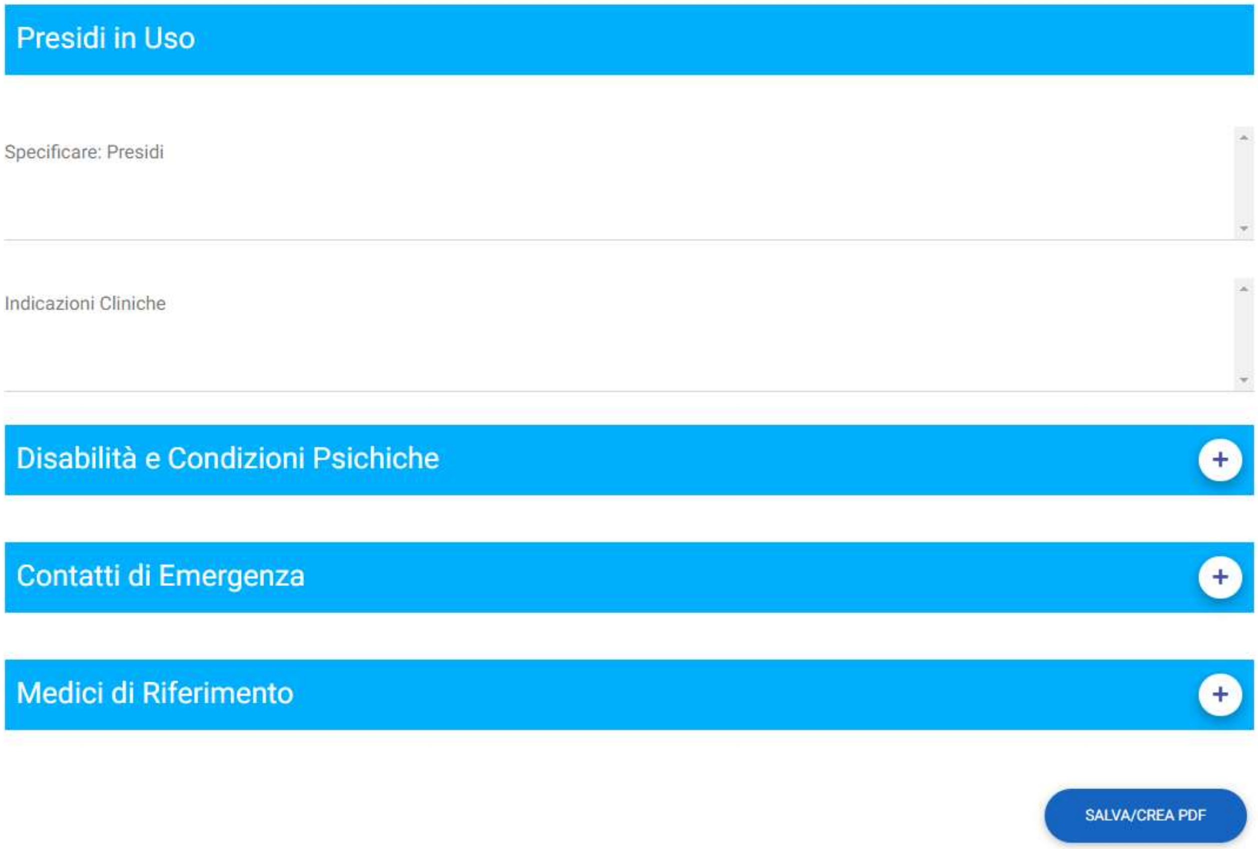

Supplement: Multimedia Appendix 1 [file humanfactors_v8i1e18587_app1.pdf]
