# Supplementary material for: A Web-Based Application for Complex Health Care Populations: User-Centered Design Approach
Source: JMIR Hum Factors. 2021 Jan 13;8(1):e18587. doi: 10.2196/18587 (PMC7840279; doi:10.2196/18587)
Supplement: Multimedia Appendix 2 [file humanfactors_v8i1e18587_app2.pdf]

Appendix Table 1. Tested *Abilita* functions

Edit, Search and Validation Functions	Change Functions	Delete Functions	Network managing Functions
Agenda	Change: Agenda	Delete: Agenda	Add patient
Allergies	Change: Allergies	Delete: Allergies	Change: Patient
Anamnesis			Delete: Patient
Anamnestic summary	Change: Anamnestic summary	Delete: Anamnesy summary	Disable HCPs personnel
Clinical visit	Change: Clinical Visit	Delete: Clinical Visit	Enable other users
Contact details	Change: contact details	Delete: contact details	Generate emergency code
Diagnosis	Change: Diagnosis	Delete: Diagnosis	Invite a person to join Abilita
Emergency	Change: Emergency	Delete: Emergency	Manage the Structure's personnel
Epicrisis	Change: Epicrisis		Manage User Registration Requests
Laboratory exams	Change: Laboratory exams	Delete: Laboratory Exams	Manage Users
Measurement	Change: Measurement	Delete: Measurement	Patient list
Medical Devices	Change: Medical Devices	Delete: Medical Devices	Save Patient
Models and certificates	Change: Models and certificates	Delete: Models and certificates	User registration requests
Notifications			Users
Nutrition	Change: Nutrition	Delete: Nutrition	
Operations	Change: Operations	Delete: Operations	
Other documents	Change: Other documents	Delete: Other documents	
Patient history	Change: Patient history	Delete: Patient History	
Physiotherapy	Change: Physiotherapy	Delete: Physiotherapy	
PsychoEducational	Change: Psychoeducational	Delete: PsychoEducational	
Psycho-functional diagnosis	Change: Psycho-functional diagnosis	Delete: Psycho functional diagnosis	
Search for			
Therapy	Change: Therapy	Delete: Therapy	
Update notifications			
Vaccines	Change: Vaccines	Delete: Vaccines	
Validate			
