# Supplementary material for: A Web-Based Application for Complex Health Care Populations: User-Centered Design Approach
Source: JMIR Hum Factors. 2021 Jan 13;8(1):e18587. doi: 10.2196/18587 (PMC7840279; doi:10.2196/18587)
Supplement: Multimedia Appendix 3 [file humanfactors_v8i1e18587_app3.pdf]

Feedback questionnaire – patients

Utility and Satisfaction

Of the functions present in the application, indicate those that are most useful for the user and his/her family, assigning a score of 1 to 5 for each item (1 = not useful at all, 5 = very useful).

Unless otherwise specified, the percentages refer to the total number of questionnaire respondents (51).

1. Is *Abilita* useful for the orderly archiving of medical documents?

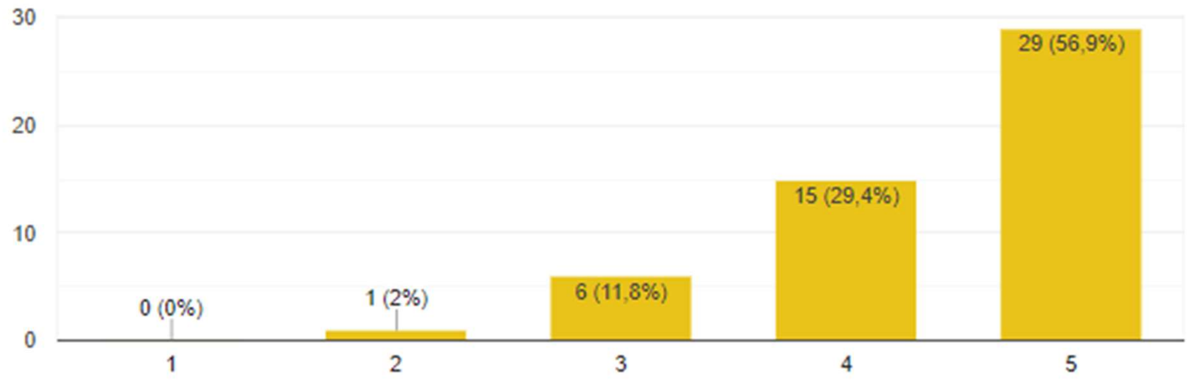

2. Is *Abilita* useful for the orderly archiving of documents concerning care and assistance?

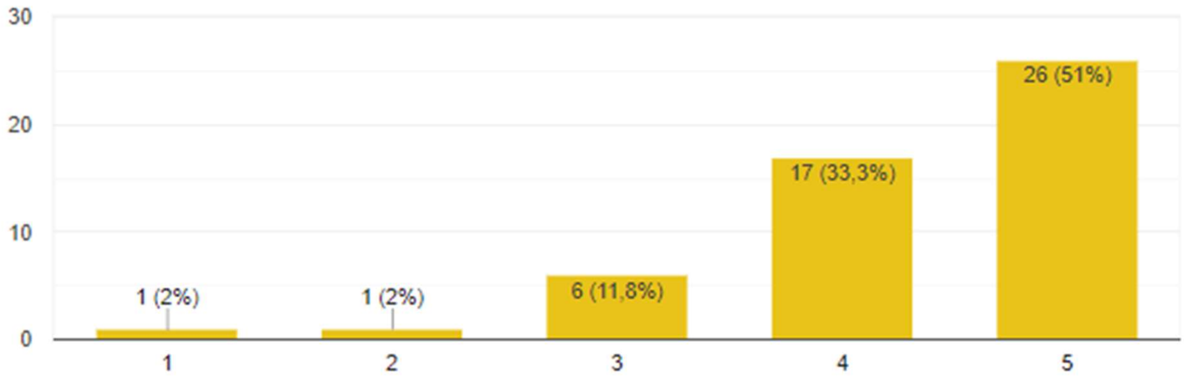

3. Is *Abilita* useful for remembering the renewal of some clinical evaluations?

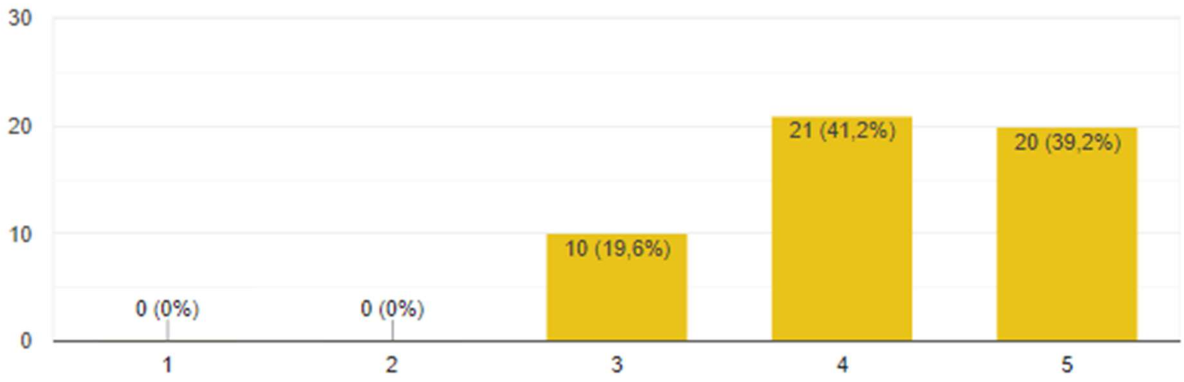

4. Is *Abilita* useful to having your medical history under control everywhere?

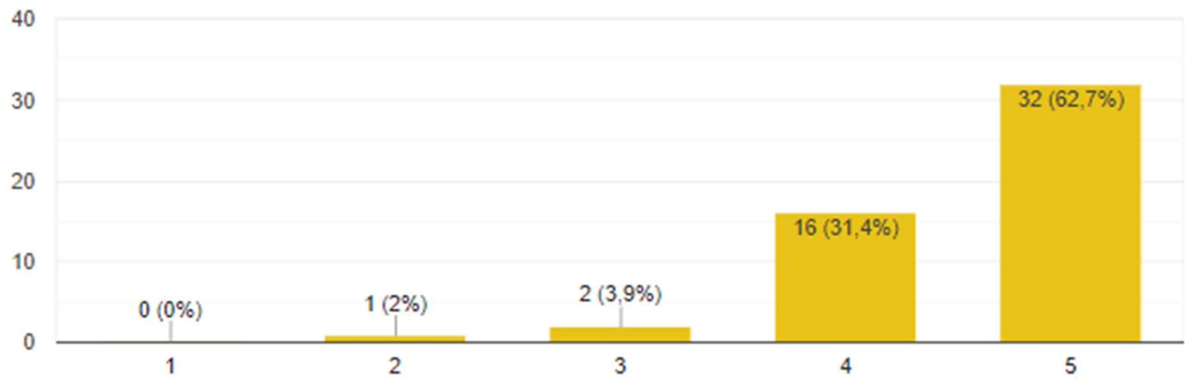

5. Does *Abilita* allow you to monitor some medical parameters when that is recommended by the HCPs?

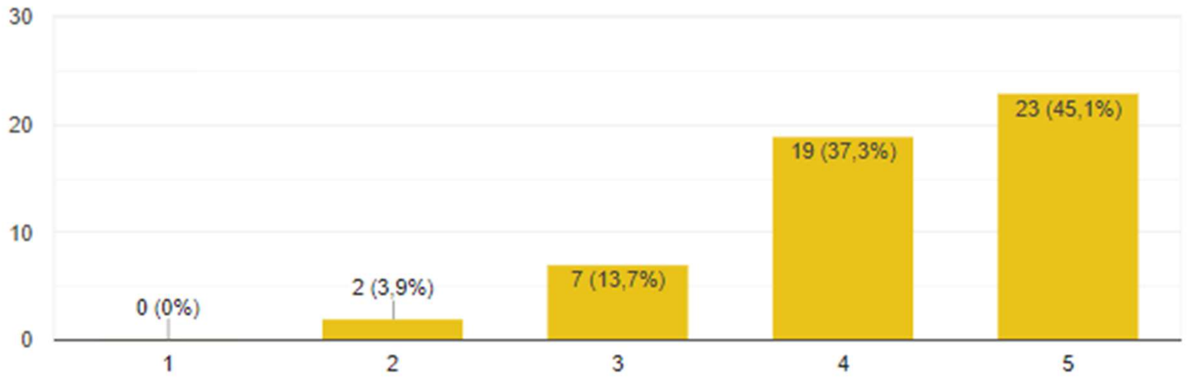

6. Is *Abilita* useful for recording daily self-measurements (e.g. blood pressure)?

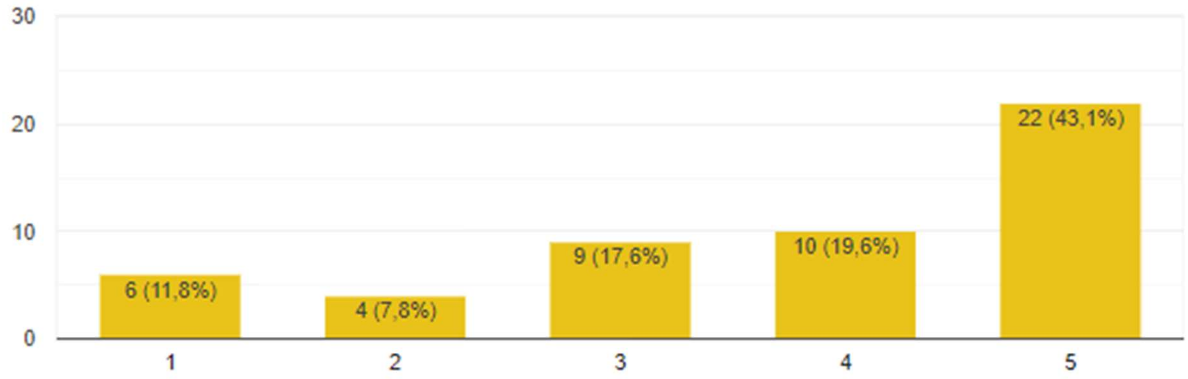

7. **Does *Abilita* allow you to share information on healthcare or psycho-educational assistance with various professionals?**

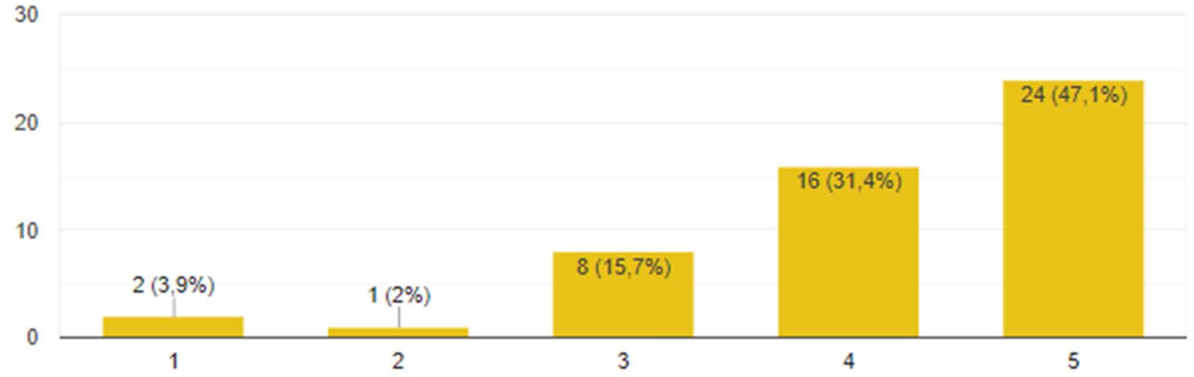

8. **Does *Abilita* allow you to receive relevant information in a health emergency away from home?**

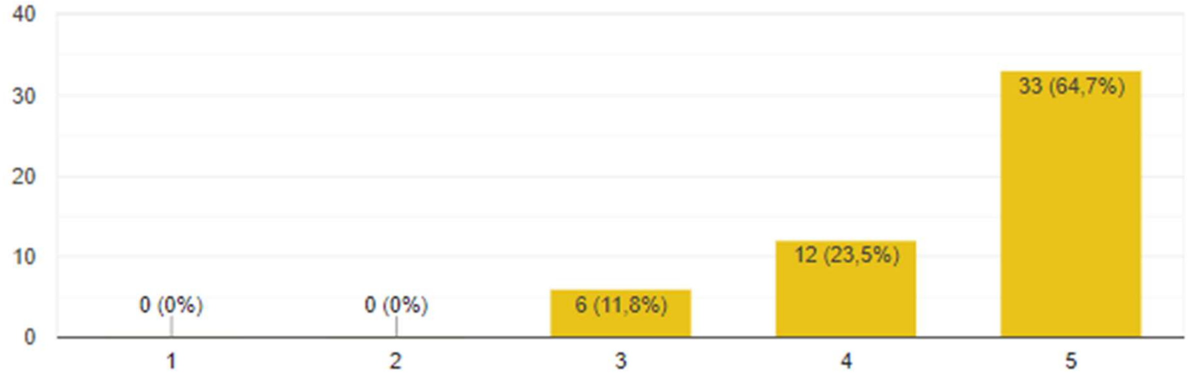

9. **Does *Abilita* allow you to share health information with HCPs without bringing your complete medical chart with you?**

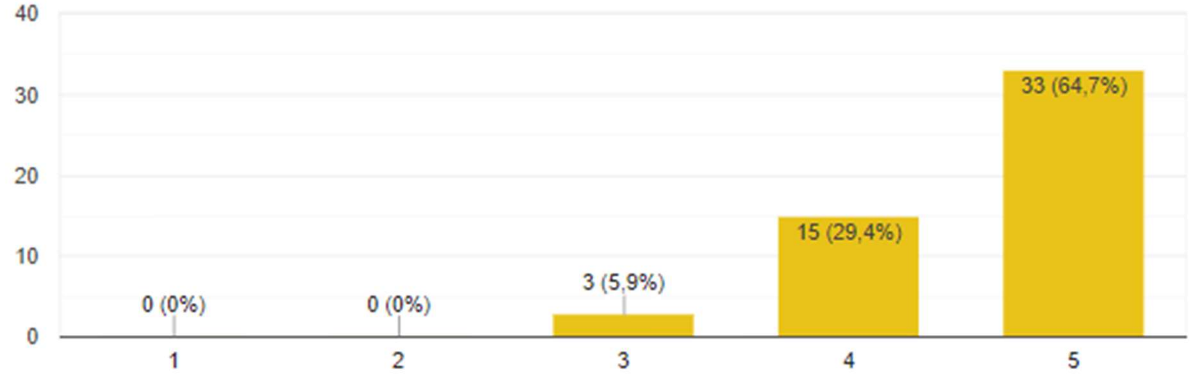

10. **Does *Abilita* help you adhere to drug therapy regimens (with reminders) and track what has actually been taken?**

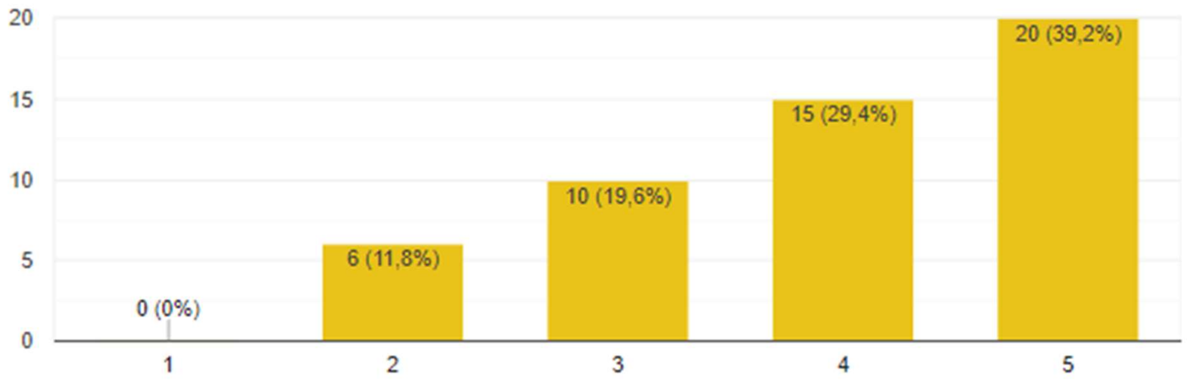

11. **Does *Abilita* help you remember what medical devices to buy/to order?**

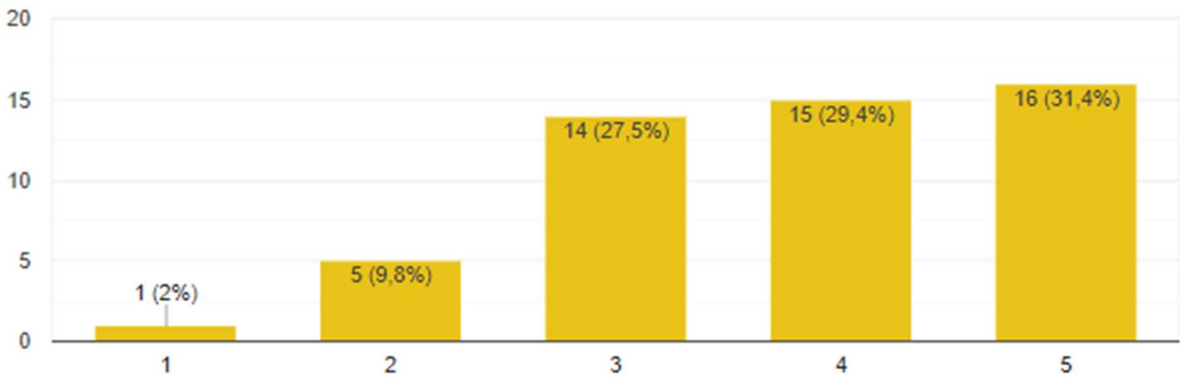

12. **Does *Abilita* help you remember administrative deadlines for requesting disability status or for other socio-procedures procedures?**

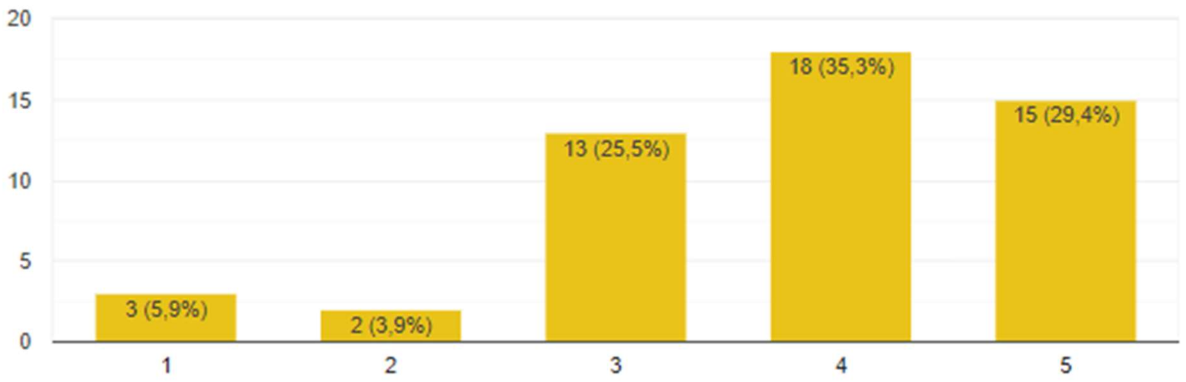

13. **Does *Abilita* help you to find a document in your archive quickly using advanced search functions?**

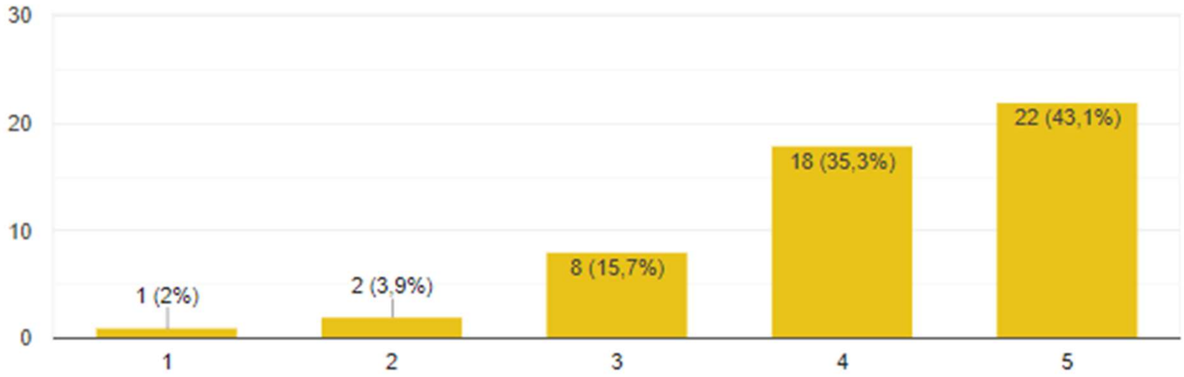

14. **Does *Abilita* provide useful information relating to bureaucratic aspects, scientific research or treatments?**

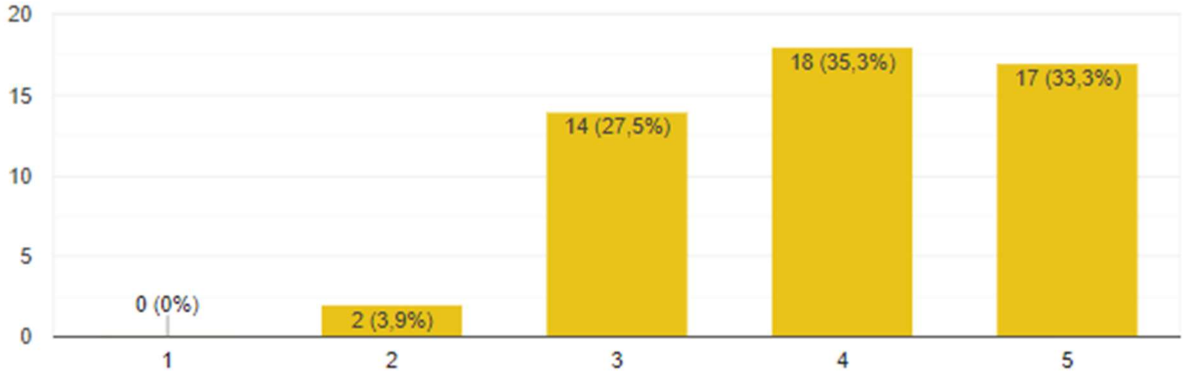

15. **Can *Abilita* support HCPs in drawing up a treatment plans and help you follow it?**

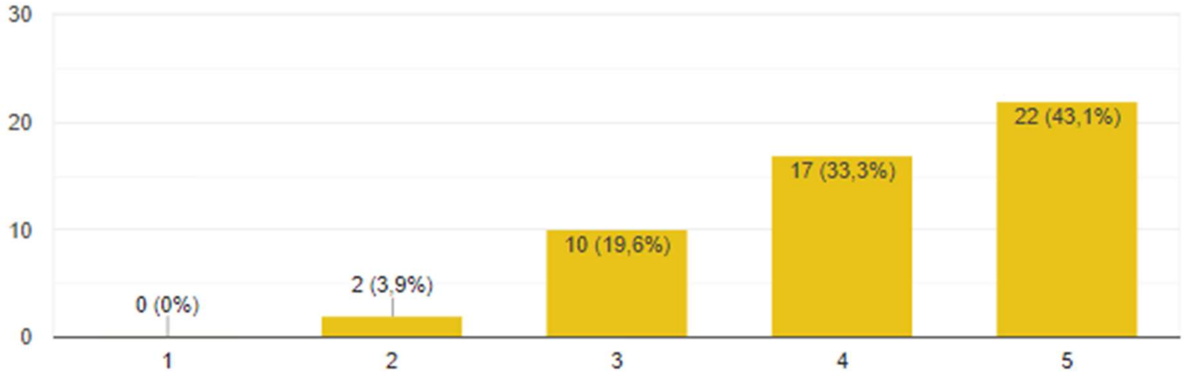

16. Overall, are you satisfied with the trial run of *Abilita*?

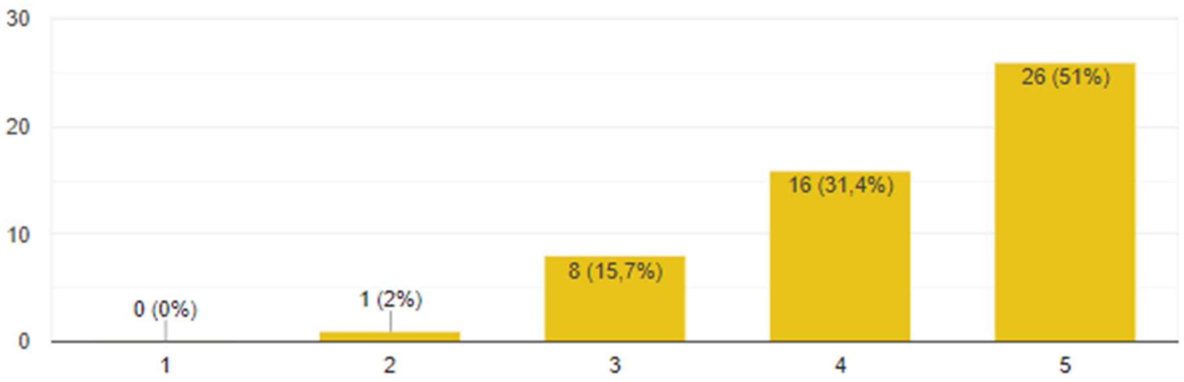

17. Within the areas currently present in *Abilita*, which functions would you implement or further articulate?

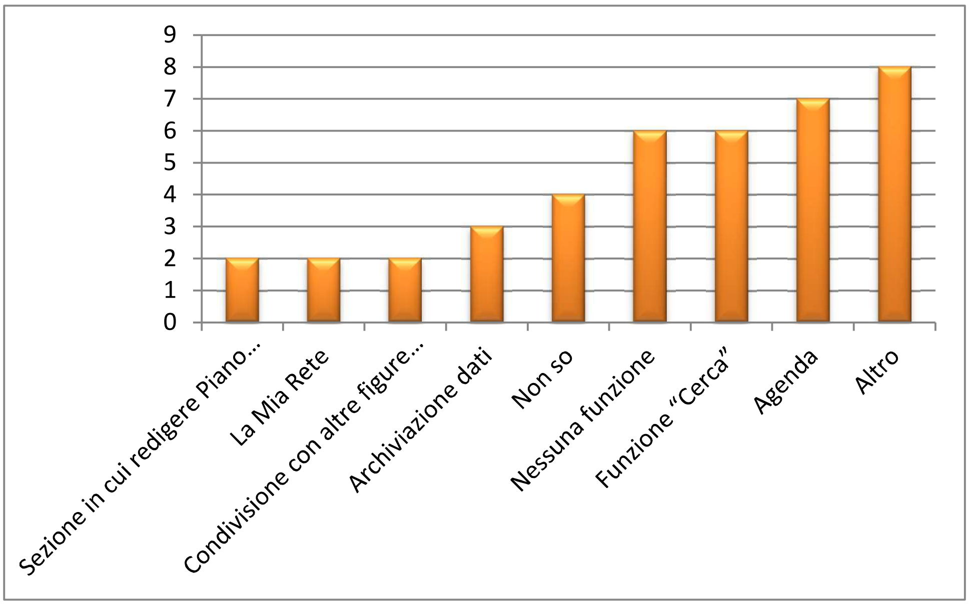

Section in which to draw up a treatment plan	2
My network	4
My data	3
None/I do not know	10
"Search" functions	6
Organizer	7
Other	9

Other – free text responses:

- The section where doctors remind the patient/caregiver about scheduled visits;
- Medical reports;
- LISA demos;
- My story;
- Bureaucratic aspects;
- My data – Administrative Forms.

18. Are there other useful functions that you think could be integrated into the platform? You can also tick more than one option.

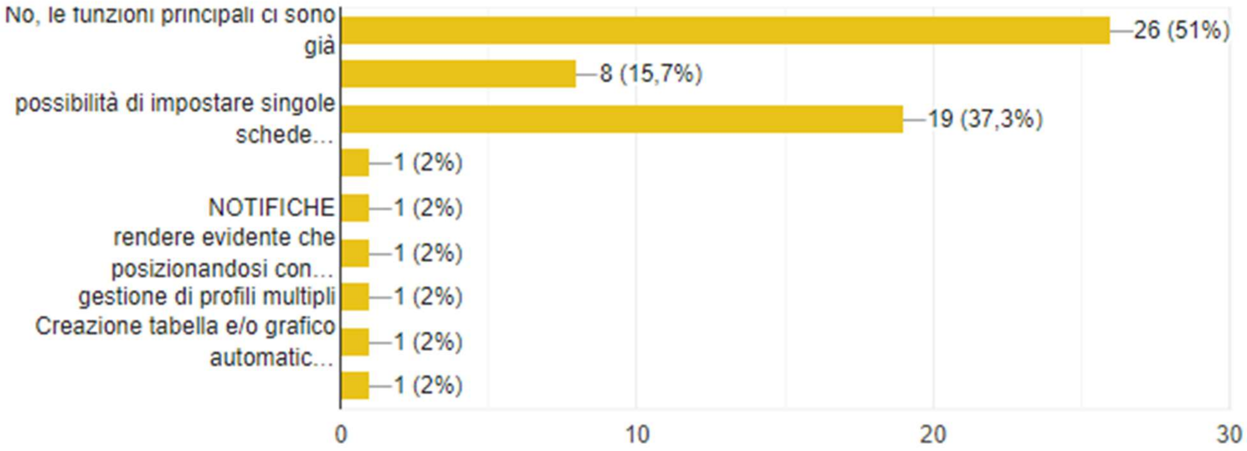

- No, the main functions are already there: 26
- Internal messaging with other users: 8
- Have the opportunity to set areas or actions as "favorites", thus creating shortcuts for the most used functions: 19

Other – free text responses:

- Section "Hospitalization" (not only for surgery): 1
- Creation of a table and/or automatic graph of a selected parameter on the basis of all its values in archived documents: 1
- management of multiple profiles: 1
- make it clearer that by positioning the cursor on the words they become "active": 1
- develop an interface where parents have direct contact with an HCP identified by the healthcare facility: 1
- Notifications: 1

Privacy and security

19. Do you think that communication of the privacy policy is sufficient? You can also tick more than one option.

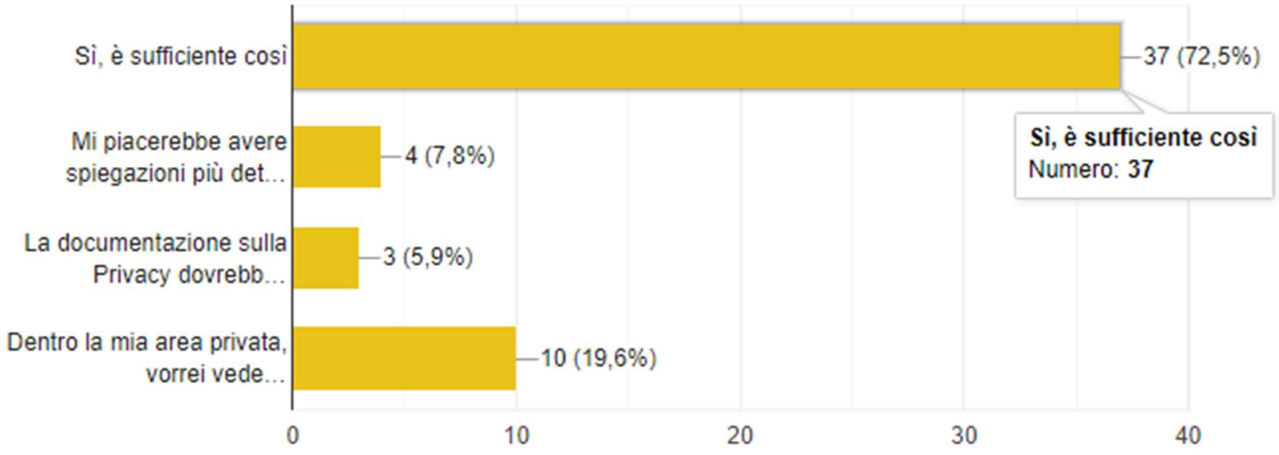

- Yes, it is sufficient: 37
- I would like to have more detailed explanations on users' profiles: 4
- Privacy documentation should be more visible on the website: 3
- Inside my private area, I would like to see and download the informed consents that I have signed: 10
- Other: 0

20. While browsing your private area of *Abilita*, did you encounter any vulnerabilities in the functioning of the system? If yes, please indicate which ones.

This question had a total of 18 answers: 13 users affirmed they did not encounter any vulnerabilities in operation; 5 users stated they had encountered the following weaknesses in their private area:

- the most difficult part was user registration
- I can't find the documentation
- I can't authorize my other parent
- Completion of the emergency card

Usability

21. Have you encountered general problems in using *Abilita*?

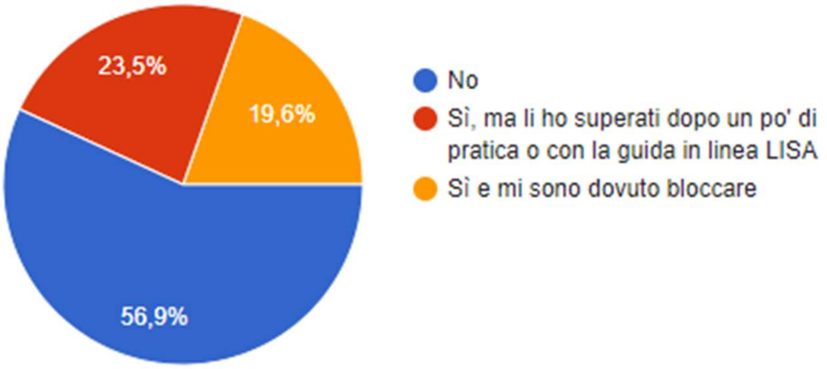

29 out of 51 respondents said they had no problem using *Abilita*; 22 respondents claimed to have encountered some problems, 10 being blocked during use while 12 overcoming them through practice or LISA online help.

22. If you have encountered problems that have blocked you, please indicate where. You can tick several options.

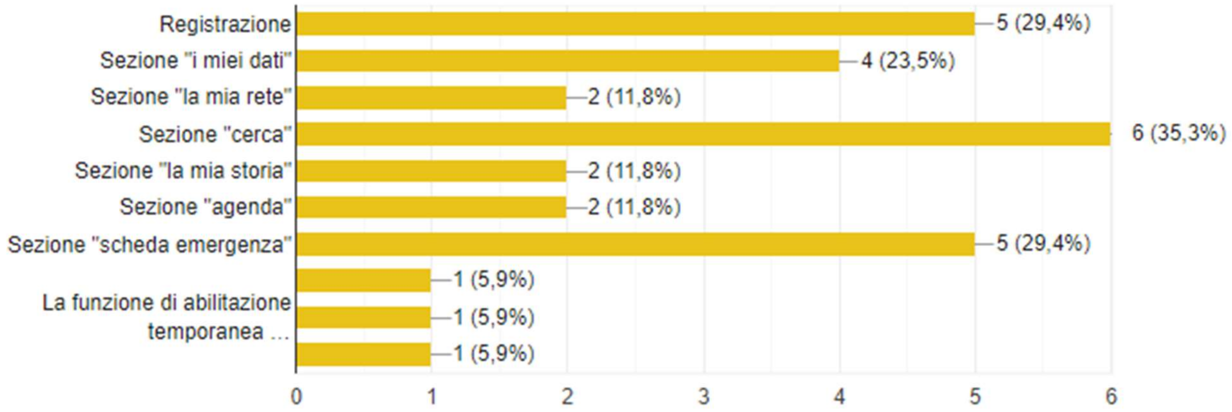

This question had a total of 17 responses.
In addition to the answers shown in the graph with more than 10% responses (registration 29%, “My data” 23%, “My network” 12%, “Search” 35%, “My story” 12%, “Organizer” 12%, “Emergency Card” 29%), 3 users reported further problems that blocked their use of *Abilita*, marking the "Other" field.

Other – free text responses:

- In the drug therapy section: the DEPAKIN ORAL SOLUTION of 40ml, 200mg / ml is missing
- The temporary authorization should be made faster.
- I had problems in selecting which profiles to authorize (perhaps it should be specified what actions each profile may do)

23. Could you easily use *Abilita* on all devices? You can tick several options.

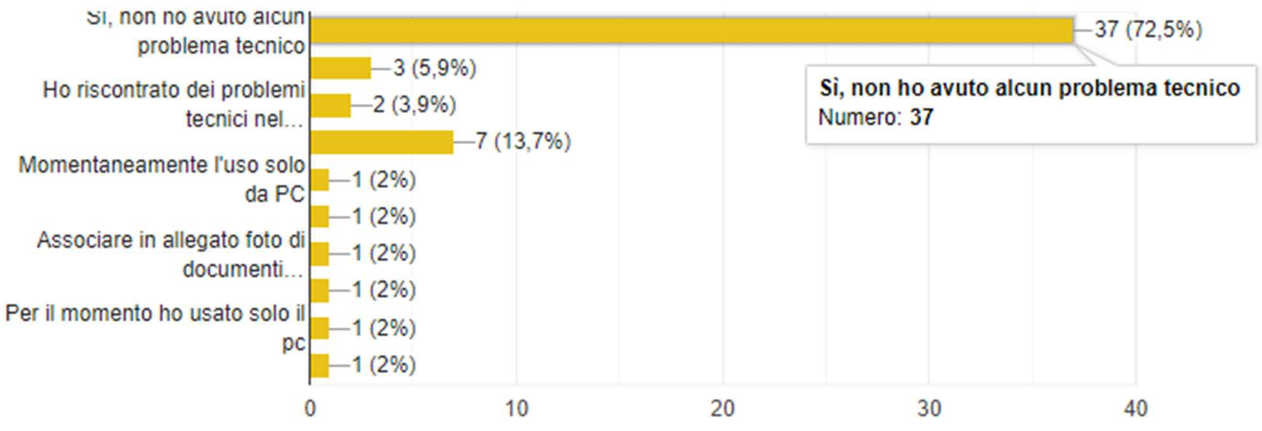

- I didn't encounter any technical problems: 37
- I encountered technical problems when using Abilita on a PC: 3
- I encountered technical problems when using Abilita on a tablet: 2
- I encountered technical problems when using Abilita on a mobile phone: 7

Other – free text responses:

- I used *Abilita* only on a PC: 3
- I haven't used it on other devices yet: 1
- I haven't used it on my mobile device yet: 1

24. When entering your data, have you ever had doubts about where to enter them?

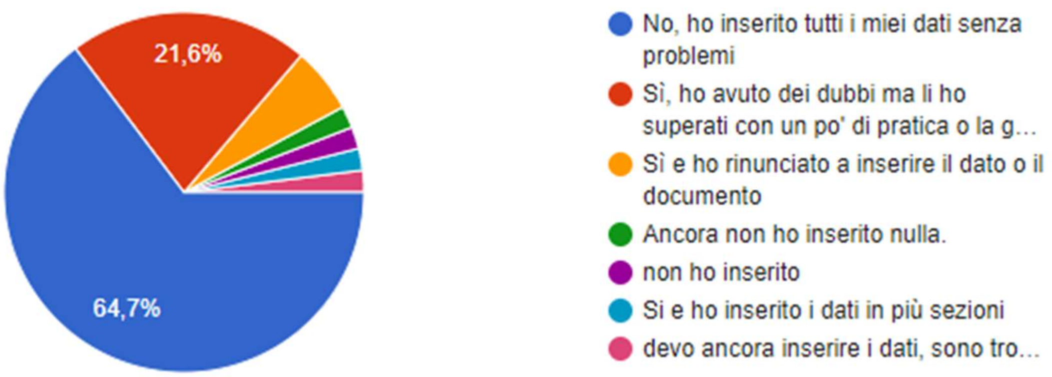

- No, I entered all my data without problems: 33 respondents
- Yes, I had some doubts, but I got over them with a little practice or the LISA online help: 12 respondents
- Yes, and I gave up entering the data or the document: 3 respondents
  3 users reported further options by filling in the "Other" field.

Other – free text responses:

- I haven't entered anything yet: 1
- I have not entered: 1
- Yes, and I entered data in several sections: 1

25. If you encountered problems in the classification of data or documents, indicate for which of them.

This question had a total of 13 responses: 7 respondents say they did not encounter any problems in the classification of data or documents in *Abilita*; 6 respondents said they encountered some classification problems in the following areas:

- Distinction between exams and checkups
- Documents related to regional and INPS certifications
- "General outline" in "My data"
- Hospital records
- Therapies
- In the My data section (Certified documents), a generic item could be added, for example: school documents, certificate of school integration, functional diagnosis etc.

26. Which user profiles do you and your family use specifically? Could you write a small outline of the profiles used, both inside and outside your family, correlating the role with the user profile? (example: mom - parent; uncle - family member; wife - caregiver; teacher - social worker; etc)?

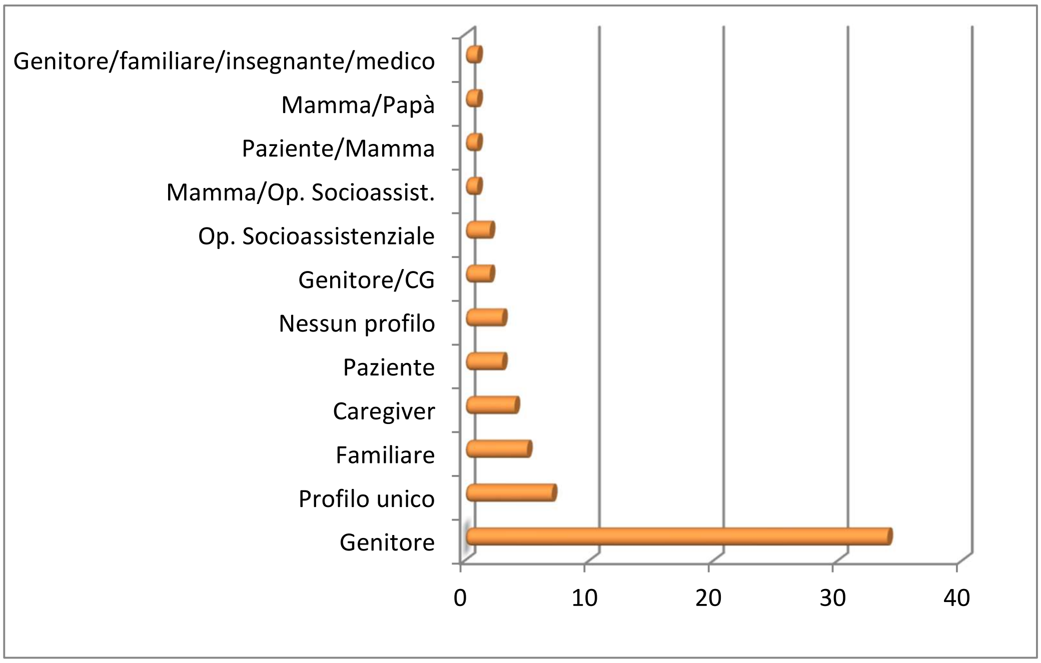

Parent	34
Unique profile	7
Familiar	5
Caregiver	4
Patient	3
No profile	3
Parent / CG	2
Social Worker	2
Mom / Social worker.	1
Patient / Mom	1
Mom Dad	1
Parent / family / teacher / doctor	1

27. Did you encounter problems using one or more user profiles? If yes, briefly describe which ones and what would you change in the profile or in its settings.

Free-text responses: 76.5% of respondents (39 users out of 51) said they had not encountered any problems using one or more profiles.
Only 7 users claim to have encountered some problems, as follows:

- Need clearer instructions 2
- Multiple profile management: 1
- Problems in inserting drugs and changes to the inserted child profile: 1
- Problems in using the "Temporary" profile: 1
- Problems entering my patient profile: 1
- Problems displaying added telephone numbers: 1

Only 4 users claim to have used only one profile.

28. What do you think works well in the profiles you used?

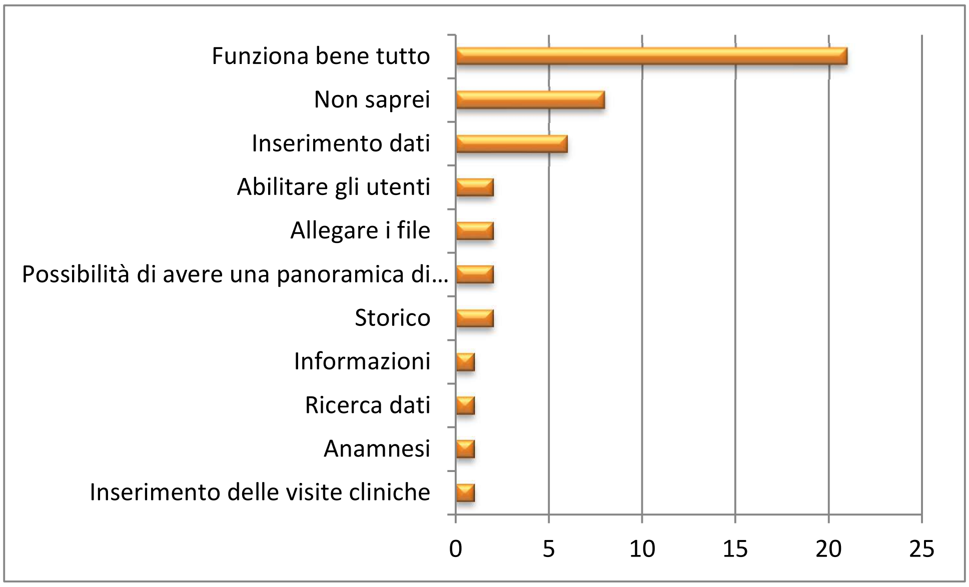

Entering clinical visits	1
Anamnesis	1
Search	1
Info Room	1
Archive	2
Possibility to have everything under control	2
Attachment of files	2
Creating my personal support network	2
Data entry	6
I do not know	8
Everything works well	21

29. Is the LISA online help always clear and complete?

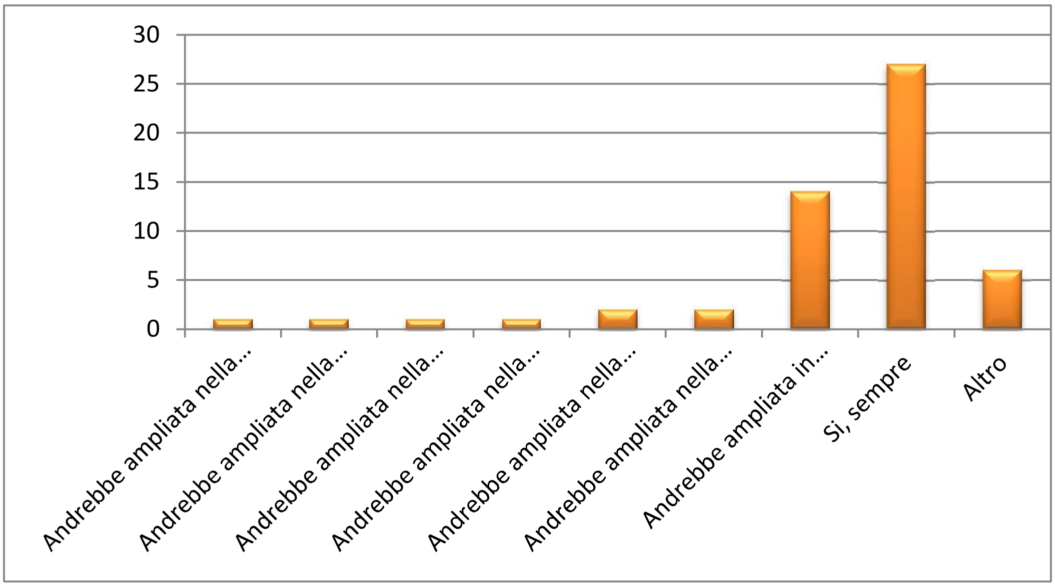

It should be implemented in the "My Network" section	1
It should be implemented in the "My Story" section	1
It should be implemented in the "Organizer" section	1
It should be implemented in the "Emergency card" section	1
It should be implemented in the "Search" section	2
It should be implemented in the "My Data" section	2
It should be implemented in all sections	14
Yes, it is always clear and complete	27
Other	6

Other – free text responses:

- I haven't tried it yet: 4
- not tested in all its parts: 1
- new sections could be introduced (e.g. CUP booking section, etc.): 1

30. **How would you like to use the *Abilita* "Organizer"? You can also tick more than one option.**

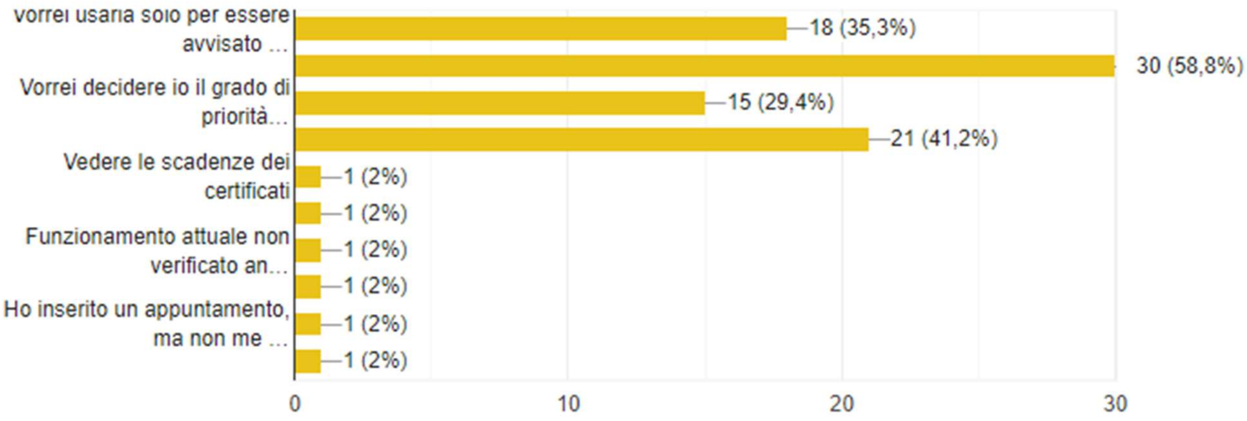

- **I would like to use it to remind me in advance of when to make visits or when to go to the offices responsible for administrative practices: 30**
- I would like to keep past events displayed so that I can see previous items month by month: 21
- I would like to be notified of appointments already booked, with equal time for reminders: 18
- I would like to decide the priority level of an event and the settings for its reminders: 15

Other – free text responses:

- It would be useful for persons who support the same patient and have so much material to bring with them
- I would like to use it to remind me to book exams/visits/renewals of treatment plans: 1
- I would like to see certificate deadlines: 1
- I do not know: 1

Other recommendations or suggestions

31. Briefly describe any technical problems encountered.

This question had a total of 24 answers.
14 users said they have not encountered any technical problems.
The remaining 10 users who answered this question report the following critical issues:

- Difficulty in entering exams
- The platform is complicated, and it takes time and effort to get to know it all
- Initial registration problem
- Cookies
- Very big problem: credentials for login, as elderly users are unfamiliar with e-mail
- Difficulty in authorization procedures
- Difficulty in having the tax code available for login
- Agenda does not report some appointments
- Lack of clarity in viewing the patient's profile and that of the doctor
- If the emergency card is not complete, the system will not allow you to save it.

32. Brief reports on security and privacy.

The question allowed the user to answer with a free text.
All respondents (23 users) said there was nothing to report about security and privacy.

33. How do you think *Abilita* can promote scientific research on your disease/disorder? What means are essential to pursuing this goal?

The question allows the user to answer with a free text.
All respondents (24 users) offered the following possibilities:

- Bringing the platform to the attention of family doctors;
- Help with research funds;
- Greater publicity;
- Elaborate statistics for each clinical pathology;
- Data export for statistical processing;
- Sharing data with caregivers can allow quick access to various solutions;
- manage, with authorization, a database capable of catch the analogies relating to the same disease;
- Through partnerships with research centers and projects;
- *Abilita* is an additional showcase for associations and for getting known by general practitioners who are experts in the sector;
- The more people join the project the more visibility will be given to the disease across the country, as the result of regional autonomy;
- Database of parameters, pre-established based on diagnosis;

- Share some data with competent staff, allowing partially free and tracked access;
- Autism: the more information you have available, including anamnesis, videos, etc., the easier it is to research the causes and the statistics.

34. Did *Abilita* facilitate relations and communication with HCPs and healthcare bodies, such as local facilities you have to contact in order, for example, to obtain a certificate or for continuity of care? (ASL, Day Centers, etc ...)?

The question allows the user to answer with a free text.
Users (34) who answered this question stated the following ideas:

- 24 participants declared with certainty that *Abilita* facilitated their communication with medical staff and centers.

In particular, users reported the following:

- *Abilita* can facilitate my relationships with the ASL-TSMREE structures - professional psychotherapy studies (Mental Health Protection and Developmental Rehabilitation)
- Yes, in order to make ASL understand the needs of families, as sometimes parents are not listened to;
- definitely yes, even if to use it at its best it takes a little practice, and I think it is not immediately usable for everyone;
- Yes, through the support of the Region and the ASL
- Absolutely yes: I believe that the goal is precisely to expand or start new dialogues with local facilities so as to build a wider network;
- It will be when ASLs are also authorized
- It will surely give me the opportunity to make all my daughter's clinical documentation available to the doctor, who does not know her, without having to carry various files with me;
- Absolutely yes. I find it a perfect way to put multiple parts together.

35. Do you think that *Abilita* can support your Association in proposing solid themes for a possible and future creation of a *PDTA* (*Diagnostic-therapeutic assistance pathway*)?

The question allows the user to answer with a free text.
Users (29) who answered this question stated the following ideas:

- 18 participants declared with certainty that *Abilita* was useful to supporting Associations for the creation of a PDTA where one was not present.

In particular, users answer:

- It could be an interesting starting point
- *Abilita* can support an individual person's PDTA and create a network of the various figures and facilities that interface with that person
- It is useful to having a single place to gather information about patients and their families

36. What are *Abilita's* strengths in your opinion? What about weaknesses?

The question allows the user to answer with a free text.
Users (28) who answered this question expressed the following ideas:
Strengths

- knowledge of one's own medical history with a click;
- Document search and monitoring;
- General project idea and how *Abilita* is organized;
- Simplicity;
- Centrality of the patient;
- Possibility of data storage and sharing;
- Useful for social inclusion by many Associations;
- Possibility of having my documents always available;
- Clinical data insertion (optimal if done directly by the doctor);
- Makes the family more united and communication with the association easier;
- Possibility for multiple figures to intervene on several fronts regarding the same patient and monitor his/her clinical history.

Weaknesses

- I think that, in addition to a direct doctor-parent channel, there should be a real-time update of the entire clinical situation;
- Little active contribution from doctors;
- Users should be more numerous to exploit the full potential of the application;
- Technology that, although it is intuitive, is not easy to use for those of an advanced age;
- Time needed to insert one’s complete history.
