# Supplementary material for: A Web-Based Application for Complex Health Care Populations: User-Centered Design Approach
Source: JMIR Hum Factors. 2021 Jan 13;8(1):e18587. doi: 10.2196/18587 (PMC7840279; doi:10.2196/18587)
Supplement: Multimedia Appendix 4 [file humanfactors_v8i1e18587_app4.pdf]

abilita

Feedback questionnaire – HCPs

Of the functions present in the *Abilita* application, individual interviews with 10 doctors and 13 nurses have been conducted to explore the app's usefulness in the follow-up of chronic patients. They were asked to indicate whether it is useful for the management of the patient's clinical data, assigning a score from 1 to 5 for each item (1 = not at all useful, 5 = very useful).

N°	Question/ Linkert score (from 1 to 5 for each item: 1 = not at all useful, 5 = very useful).	Not useful score 1 or 2	Enough score 3	Very useful score 4 or 5
1	Is *Abilita* useful for the orderly archiving of patient's medical records?	0	6 (26%)	17 (74%)
2	Is *Abilita* useful for remembering the renewal of some clinical evaluations?	0	9 (39%)	14 (61%)
3	Is *Abilita* useful to easily have the clinical history of patients under control?	0	3 (13%)	20 (87%)
4	Does *Abilita* help to monitor medical parameters according to previous recommendations?	3 (13%)	3 (13%)	17 (74%)
5	Does *Abilita* allow you to share information with other professionals?	0	8 (35%)	15 (65%)
6	Does *Abilita* allow you to have relevant information in case of health emergency?	2 (9%)	8 (35%)	13 (56%)
7	Is *Abilita* useful to have the documents when the patient does not have them with him/her?	0	2 (9%)	21 (91%)
8	Does *Abilita* help to find a document in patients archive quickly using advanced search functions?	3 (13%)	4 (17%)	16 (70%)
9	Does *Abilita* provide useful information relating to bureaucratic aspects, scientific research or treatments?	2 (9%)	10 (43%)	11 (48%)
10	Can *Abilita* support you in drawing up a treatment plan?	1 (4%)	8 (35%)	14 (61%)
11	*Could you easily use Abilita on all devices?*	3 (13%)	3 (13%)	17 (74%)
12	*Did you encountered problems in the classification of data / documents?*	1 (4%)	4 (17%)	18 (78%)
13	Did you think *Abilita* can promote scientific research on your professional goals?	4 (18%)	6 (26%)	13 (56%)
14	Did *Abilita* facilitate relations and communication between HCPS?	0	4 (18%)	19 (82%)
15	Was the *Abilita* interface usable for you?	0	3 (13%)	20 (87%)
16	Overall, are you satisfied with the trial run of *Abilita*?	0	2 (9%)	21 (91%)

The percentages refer to the total number of respondents to the questionnaire (23).
